# African swine fever virus histone-like protein A104R subverts host antiviral defenses by suppressing the nuclear translocation of IRF3 and STAT1

**DOI:** 10.1186/s13567-026-01874-z

**Published:** 2026-11-06

**Authors:** Nuwan Gamage, Pramodya Rathnapala, Ji-Won Cha, D. K. Haluwana, Ashan Subasinghe, Asela Weerawardhana, Jong-Soo Lee

**Affiliations:** 1 College of Veterinary Medicine, Chungnam National University, Daejeon, Republic of Korea; 2 Department of Biosystems Technology, Faculty of Technology, University of Ruhuna, Kamburupitiya, Matara, Sri Lanka

**Keywords:** African swine fever virus (ASFV), A104R, KPNA, IRF3, STAT1, Type I interferon

## Abstract

A104R of African swine fever virus (ASFV), putative histone-like protein, is known to be involved in essential processes such as viral DNA replication, repair, and transcription. Although it has been revealed that the deletion of A104R affects ASFV pathogenicity for live attenuated vaccine development, the molecular mechanism underlying those effects remains unclear. Here, we report a novel molecular mechanism of A104R that suppresses karyopherin-α (KPNA) mediated antiviral responses. A104R competitively bound to the major nuclear localization signal (NLS) region on KPNA3 and KPNA4, thereby disrupting their interaction with interferon regulatory factor 3 (IRF3). A104R also bound to the unique C terminal nonclassical NLS region of KPNA6 and competitively inhibited the interaction between KPNA6 and phosphorylated signal transducer and activator of transcription 1 (STAT1). Therefore, A104R inhibited the nuclear translocation of IRF3 and phosphorylated STAT1, thereby suppressing type I interferon (IFN) signalling and IFN-mediated antiviral responses. Consequently, ectopic expression of A104R enhanced DNA virus replication and suppressed IFN production by inhibiting the IRF3-mediated type I IFN signalling pathway and suppressed the interferon-β (IFN-β)-mediated antiviral responses. Taken together, these results highlight that A104R plays a dual role in suppressing IFN response and IFN-mediated antiviral responses. This mechanism provides new insight into the A104R induced ASFV pathogenesis, which provide a critical implication for a better understanding of the ASFV pathogenesis.

**Supplementary Information:**

The online version contains supplementary material available at https://doi.org/10.1186/s13567-026-01874-z.

## Introduction

African swine fever (ASF) is a devastating viral disease affecting both domestic and wild pigs that causes significant economic losses [1]. Acute African swine fever virus (ASFV) infection in domestic pigs can result in mortality rates of up to 100%, often accompanied by high fever, hemorrhages, and multiple organ failure [2]. ASFV is a large, enveloped icosahedral arbovirus and the sole member of the family *Asfarviridae* [3]. Its genome comprises a linear, nonsegmented, double-stranded DNA ranging from 170 to 193 kilobase pairs and encoding more than 150 proteins involved in immune evasion, transcription, replication, structural assembly, and host cell remodeling [4]. Currently, only three licensed vaccines are available in Vietnam [5].

ASFV-encoded proteins evade the host immune responses to promote viral replication and pathogenesis [6]. During DNA virus infection, pathogen-associated molecular patterns (PAMPs), such as viral DNA, are recognized by host pattern recognition receptors (PRRs), activating a cascade of innate immune responses [7]. In particular, cyclic GMP–AMP synthase (cGAS) recognizes cytoplasmic double-stranded DNA and catalyzes the synthesis of cyclic GMP–AMP dinucleotide (2′,3′-cGAMP) [8]. Synthesized cGAMP activates the signal pathway to the stimulator of interferon (IFN) genes (STING), TANK-binding kinase 1 (TBK1), and IFN regulatory factor 3 (IRF3). The transcriptional factor IRF3 is phosphorylated (PY-IRF3) by TBK1 to form a dimer. The PY-IRF3 dimer then translocates into the nucleus, facilitated by a subset of nuclear transport receptors in the karyopherin-α (KPNA) family, where it drives type I IFN production [9].

After synthesis and release, type I IFN binds to IFN receptors and initiates the activation of the Janus kinase (JAK)-signal transducer and activator (STAT) signaling pathway [10]. Upon IFN-α/β receptor engagement, JAK1 and tyrosine kinase 2 (TYK2) phosphorylate signal STAT1 and STAT2, forming homo- or hetero-dimers via reciprocal SH2-domain phosphotyrosine interactions [11]. Finally, the IFN-stimulated gene (ISG) factor 3, a heterotrimeric complex, is subsequently formed through association with interferon regulatory factor 9 (IRF9). KPNA mediates the nuclear import of phosphorylated STAT1 (PY-STAT1), allowing it to bind to IFN-stimulated response elements (ISRE) or IFN-γ-activated sites (GAS), thereby inducing ISG expression that confers an antiviral state [12]. Given that the type I IFN response serves as a rapid and robust antiviral defense mechanism in hosts [13], almost all viruses have evolved diverse strategies to block or suppress IFN secretion, disrupt IFN-mediated signaling pathways, and inhibit ISG function [14]. However, the functions and host-interaction mechanisms of many ASFV proteins remain incompletely understood, particularly those with established roles in viral genome biology.

ASFV A104R is a small highly conserved, putative histone-like protein (pA104R) [15]. The structure of pA104R has been successfully characterized, revealing structural similarity to eukaryotic histones and homologous to members of the bacterial HU/IHF family of histone-like proteins, sharing sequence and structural features [16–19]. Notably, A104R is the only histone-like protein known to be encoded by a eukaryotic virus. Functionally, A104R binds to both single- and double-stranded DNA in an ATP-independent manner under a wide range of conditions. The protein is expressed during the late phase of infection and localizes to both the cell nucleus and cytoplasmic viral DNA replication sites [20, 21].

A recent in vivo study demonstrated that deletion of A104R from the virulent Georgia isolate drastically reduced viral virulence in domestic pigs, highlighting its critical role in the ASFV pathogenesis [22]. A104R has been implicated in essential processes such as viral DNA replication, repair, recombination, and transcription [21]. It may also regulate nucleoid compaction and progeny virion assembly. Nonetheless, the biological role of A104R in modulating host–virus interactions remains largely unknown.

In this study, we identified a novel immune evasion mechanism mediated by A104R, highlighting its role in regulating the type I IFN signaling pathway and our results provide new insights into the sophisticated strategies employed by ASFV to suppress host antiviral defenses.

## Materials and methods

### Cells and antibodies

HEK293T cells (ATCC^®^ CRL-11268™), 293-Dual™ hSTING-A162 cells (Invivogen, 293d-a162), PK-15 cells (ATCC^®^ CCL-33), A549 cells (ATCC CCL-185), Vero cells (ATCC^®^ CCL-81™), and monkey kidney epithelial cells (MA-104) cells were cultured in Dulbecco’s modified Eagle medium (DMEM)-high glucose (Cytiva). The 3D4/21 cells (ATCC^®^ CRL2843™) and primary porcine alveolar macrophages (primary PAMs) (Optipharm Inc., Republic of Korea) were cultured in supplemented RPMI (Hyclone; Cytiva). Cells were supplemented with 10% fetal bovine serum (FBS) (Gibco) and 1% antibiotic/antimycotic (Gibco) or 1% penicillin–streptomycin (Gibco) and incubated at 37 °C in a humidified atmosphere containing 5% CO_2_. Antibodies used for immunoblotting and immunoprecipitation analysis are listed in Table 1 and were used according to the manufacturer’s instructions.

**Table 1:** **Antibodies used in this study**

Reagent	Target	Host and clonality	Vendor	Catalog no.	Application and dilution
Antibody	Flag	Mouse monoclonal	Cell Signaling Technology	8146	IB/IP 1:3000
Antibody	Strep	Mouse monoclonal	Qiagen	34,850	IB/PD 1:3000
Antibody	GST	Mouse monoclonal	Santa Cruz Biotechnology	sc-138	IB/PD 1:3000
Antibody	IRF3	Rabbit monoclonal	Cell Signaling Technology	4302	IB/IP 1:1000
Antibody	p-IRF3	Rabbit monoclonal	Cell Signaling Technology	4947	IB 1:1000
Antibody	TBK1	Rabbit monoclonal	Cell Signaling Technology	3504	IB 1:1000
Antibody	p-TBK1	Rabbit monoclonal	Cell Signaling Technology	5483	IB 1:1000
Antibody	STAT1	Rabbit monoclonal	Cell Signaling Technology	9175	IB 1:1000
Antibody	p-STAT1 (Y701)	Rabbit monoclonal	Cell Signaling Technology	9167	IB 1:1000
Antibody	NF-κB p65	Rabbit monoclonal	Cell Signaling Technology	8242	IB 1:1000
Antibody	p-NF-κB p65	Rabbit monoclonal	Cell Signaling Technology	3033	IB 1:1000
Antibody	IκBα	Rabbit monoclonal	Cell Signaling Technology	9242	IB 1:1000
Antibody	p-IκBα	Rabbit monoclonal	Cell Signaling Technology	2859	IB 1:1000
Antibody	V5	Rabbit monoclonal	Bethyl Laboratories	2859	IB 1:1000
Antibody	KPNA3	Mouse monoclonal	Santa Cruz Biotechnology	sc-514101	IB/IP 1:1000
Antibody	KPNA4	Mouse monoclonal	Santa Cruz Biotechnology	sc-390535	IB/IP 1:1000
Antibody	KPNA6	Rabbit polyclonal	Abclonal	A7363	IB/IP 1:1000
Antibody	β-Actin	Mouse monoclonal	Santa Cruz Biotechnology	sc-47778	IB 1:3000
Antibody	Lamin B1	Mouse monoclonal	Santa Cruz Biotechnology	sc-20682	IB 1:1000
Antibody	α-Tubulin	Mouse monoclonal	Santa Cruz Biotechnology	sc-8035	IB 1:1000

IB, immunoblotting; IP, immunoprecipitation; PD, pulldown; p-, phosphorylated; IRF3, interferon regulatory factor 3; TBK1, TANK-binding kinase 1; STAT1, signal transducer and activator of transcription 1; NF-κB, nuclear factor κB; IκBα, inhibitor of κB alpha; KPNA, karyopherin-α; β-actin, beta-actin; α-tubulin, alpha-tubulin

### Plasmids

The complete coding sequence of A104R from the ASFV genome (GenBank accession no. FR682468.1) was amplified by polymerase chain reaction (PCR) and cloned into the pIRES-Flag, pEXPR-Strep, and pEGB-GST expression vectors to generate Flag-tagged, Strep-tagged, and GST-tagged A104R constructs, respectively. The interferon-β (IFN-β) promoter reporter and luciferase reporter plasmids were constructed as described previously [23]. The pIRES-3×Flag-cGAS plasmid was kindly provided by Dr Jae U. Jung (Department of Cancer Biology, Lerner Research Institute, Cleveland Clinic). The coding sequences of human stimulator of interferon genes (STING), TANK-binding kinase 1 (TBK1), interferon regulatory factor 3 (IRF3), inhibitor of nuclear factor kappa B kinase subunit epsilon (IKKε), and signal transducer and activator of transcription 1 (STAT1) were amplified by PCR from the corresponding template DNAs and cloned into the pIRES-Flag vector to generate N-terminal Flag-tagged expression constructs. Similarly, the coding sequences of human KPNA1, KPNA2, KPNA3, KPNA4, KPNA5, and KPNA6 were amplified by PCR and inserted into the pIRES-Flag vector to generate Flag-tagged KPNA expression plasmids.

For domain-mapping experiments, full-length KPNA4 and KPNA6 and their corresponding truncation constructs were amplified by PCR and cloned into the pEGB-GST vector to generate GST-tagged fusion proteins. All plasmid constructs were verified by sequencing (Bionics, Korea).

### ASFV A104R stably expressed cell line generation

A104R stably-expressing 3D4/21 cells, and MA-104 cells were produced by transfecting the A104R-pIRES-Flag construct using Lipofectamine 2000 (Invitrogen). Following transfection, cells were selected with puromycin. Expression of the A104R was verified by immunoblotting with Flag antibody.

### Virus infection and plasmid transfection

Green fluorescent protein (GFP)-expressing adenovirus (ADV-GFP) was propagated in the PK-15 cells. Herpes simplex virus (HSV-GFP) and Vaccinia virus (VACV-GFP) were propagated in the Vero cells and titrated by plaque assay [24]. The virus was introduced to the target cells using DMEM containing 1% FBS at a specific multiplicity of infection (MOI). After incubating for 2 h at 37 °C, cell-free viruses were removed and replaced with fresh DMEM enriched with 10% FBS. At the specified times, cells along with supernatants were collected and centrifuged at 3000 rpm for 3 min. To perform Enzyme-Linked Immunosorbent Assay (ELISA), the supernatant was collected from each sample. The cell pellet was subsequently resuspended in 300 µL of phosphate-buffered saline (PBS), and the fluorescence of each sample was measured (Glomax detection system, Promega). Plasmids were transfected into PK-15, 3D4/21, and MA-104 cells with Lipofectamine 2000 (Invitrogen) and HEK293T and 293-Dual™ hSTING-A162 with polyethyleneimine (PEI; Polyscience Inc.23966) following the manufacturer’s protocol.

### Virus titer determination

Cells infected with ADV-GFP, HSV-GFP, and VACV-GFP, along with their culture supernatants, were collected at specified intervals to determine virus titers (plaque assays on A549 or Vero cells). A monolayer of these cells was seeded in 12-well culture plates for a 12 h incubation period. Cells were then exposed to a series of diluted virus-containing supernatants in 1% DMEM for 2 h. Following the incubation, the inocula were removed and replaced with DMEM mixed with 0.1% agarose (Sigma-Aldrich). The plates were then incubated at 37 °C for an additional 36 h and observed for plaque development under microscope (200 × magnification). Virus titers were calculated based on the number of plaque-forming units (PFU) and the dilution factor.

### A104R transcription assay

Primary PAMs were infected with ASFV Korea/wild boar/Hwacheon/2020–2287 isolate (GenBank accession no. OR159219.1) at an MOI of 0.5. Samples of cell pellets were taken at intervals of 0, 3, 6, 9, 12, 15, and 18 hours post-infection (hpi). Total RNA was then extracted, and quantitative real-time PCR (RT-qPCR) was conducted using A104R-specific primers (Table 2).

**Table 2:** **Primers used for real-time quantitative PCR**

Target gene	Forward primer (5′–3′)	Reverse primer (5′–3′)	AT (°C)
pIFN-β	AAATCGCTCTCTGATGTGT	TGCTCCTTTGTTGGTATCG	62
pIFN-γ	CCATTCAAAGGAGCATGGAT	GAGTTCACTGATGGCTTTGC	63
pIL-6	CACCGGTCTTGTGGAGTTTC	GTGGTGGCTTTGTCTGGATT	66
pIL-1β	GGGACTTGA AGAGAGAAGTGG	CTTTCCCTTGATCCCTAAGGT	65
pTNFα	TCACAGGGCAATGATACCC	GGGGATCCTGCCCTGTGA	66
pMCP-1	CAGAAGAGTCACCAGCAGCA	TCCAGGTGGCTTATGGAGTC	67
pMX-1	TAGGCAATCAGCCATACG	GTTGATGGTCTCCTGCTTAC	62
pISG-15	AGCATGGTCCTGTTGATGGTG	CAGAAATGGTCAGCTTGCACG	63
pIFIT1	CTGACTCACAGCAACCATG	CTTTCAGGTGTTTCACATAGG	62
pIFITM3	GTCGTCTGGTCCCTGTTCAAC	GAGTAGGCGAAAGCCACGAA	68
pOASL	TCCCTGGGAAGAATGTGCAG	CCCTGGCAAGAGCATAGTGT	68
pPKR	ATTGCGAGAAGGTAGAGCGT	TTCCATTTGGTGAAAGGCAC	65
A104R	GGAATTCCATATGATGTCGACAAAAAAAAAGCCCACAATTA	TCCGCTCGAGATTTAACATATCATGAACAGGTTTCAATGC	72

AT, annealing temperature; p, porcine; IFN, interferon; IL, interleukin; TNF, tumor necrosis factor; MCP-1, monocyte chemoattractant protein 1; MX-1, MX dynamin-like GTPase 1; ISG15, interferon-stimulated gene 15; IFIT1, interferon-induced protein with tetratricopeptide repeats 1; IFITM3, interferon-induced transmembrane protein 3; OASL, 2′-5′-oligoadenylate synthetase 1; PKR, protein kinase R

### RNA interference experiments

The small interfering RNA (siRNA) specifically designed to target A104R (siA104R) was created and produced by Bioneer^®^ in Korea. A nontargeting siRNA (siControl) served as the negative control. Primary PAMs seeded in a 24-well plate (1 × 10^6^ cells per well) were transfected with either siControl or siA104R using RNAimax (Inviogen). At 6 hours post-transfection (hpt), cells were either left uninfected or infected with ASFV Korea/wild boar/Hwacheon/2020–2287 isolate (GenBank accession no. OR159219.1) at an MOI of 0.5. The mRNA expression levels of the target genes were measured using RT-qPCR. All experiments related to ASFV infection were conducted in accordance with the Standard Operating Procedure (SOP) in the biosafety level 3 (BSL-3) laboratory of the National Institute of Wildlife Disease (NIWDC) in Korea.

### ELISA

ELISA was performed to detect secreted pro-inflammatory cytokines in cell culture supernatants. Porcine IFN-β (CUSABIO, CSB-#E09890p) and interleukin (IL)-6 (CUSABIO, CSB- #E06786p), human IL-6 (BD OptEIA, #5,552,220), and human IFN-β (CUSABIO, #CSB-E09889h) were used for analysis according to the manufacturer’s protocols.

### Quantitative real-time PCR

PK-15 or 3D4/21 cells, either transfected with Flag-A104R or a Flag vector as a control plasmid, were cultured in six-well culture plates (1 × 10^6^ cells/well) and maintained at 37 °C. These cells were then infected with ADV-GFP (MOI = 1). Upon 0, 12, and 24 hpi, harvested cells were subjected to total RNA extraction using Machery-Nagel NucleoSpin RNA kit (790955.250), and complementary DNA (cDNA) was synthesized. The cDNA levels were measured through RT-PCR using the Smart Gene SYBR Green Q-PCR master mix (SG. SYBR.500) kit, following the manufacturer’s guidelines. All primer sequences used in these experiments are detailed in Table 2.

### Protein immunoprecipitation

Plasmids were cotransfected, and cells were harvested at 36 hpt. For pull-down assay or immunoprecipitation (IP) whole-cell lysates (WCLs) were prepared by lysing the cells with protease and phosphatase inhibitor cocktail (Sigma) in a radio-immunoprecipitation assay (RIPA) lysis buffer containing 50 mM Tris–HCl, 150 mM NaCl, 0.5% sodium deoxycholate, 1% IGEPAL, 1 mM NaF, and 1 mM Na_3_VO_4_, followed by sonication (30% amplitude, 10 s, three cycles) using a sonicator (Sonics). The lysates were then centrifuged at 12,000 rpm at 4 °C for 10 min. WCLs were mixed in a 1:1 ratio with sample buffer (Sigma; S3401). The obtained samples were then heated at 100 °C for 10 min and subjected to sodium dodecyl sulfate–polyacrylamide gel electrophoresis (SDS-PAGE). For IP, cell lysates were precleared with Sepharose 6B (GE Healthcare Life Science) for 3 h at 4 °C. The lysates collected were subsequently incubated overnight at 4 °C with bait protein specific antibody, or glutathione-linked Sepharose (GST) beads (Amersham Biosciences), or Strep beads (IBA Life Sciences). Proteins attached to antibodies were immunoprecipitated (IP) with protein A/G PLUS-agarose (SC-2003; Cell signaling) beads. The beads were gathered by centrifugation and subsequently washed with lysis buffer. Precipitates were then analyzed using SDS-PAGE followed by immunoblotting with the relevant antibodies.

### Immunoblot analysis

Cells that were harvested underwent lysis and obtained whole cell lysates (WCL), IP samples were then separated using SDS-PAGE and then transferred onto a polyvinylidene difluoride (PVDF) membrane using a Trans-Blot^®^ semi-dry transfer cell (Bio-Rad). Subsequently, the membrane was blocked for 1 h in a solution containing 5% bovine serum albumin (BSA) and then incubated overnight at 4 °C with the primary antibody. On the following day, membranes were washed with Tris-buffered saline with tween-20 (TBST) or phosphate-buffered saline with Tween-20 (PBST), followed by a 2 h incubation at room temperature with a horseradish peroxidase-conjugated (HRP) secondary antibody. Then membranes were washed and visualized using an enhanced chemiluminescence detection system (ECL-GE Healthcare, Little Chalfont, United Kingdom) using a Las-3000 mini Lumino Image Analyzer.

### Luciferase reporter assay

HEK293T and hSTING cells were grown in 12-well plates (3.5 × 10^5^ cells/well). These cells were then incubated at 37 °C in a 5% CO_2_ for 12 h. The hSTING cell line, which has been overexpressed with human STING, was utilized to assess the activation of IFN-β luciferase activity by poly (dA:dT) cGAS and 2′3′-cGAMP.

HEK293T cells were used to investigate the IFN-β luciferase reporter activity triggered by STING, TBK1, IRF3, and IKKε. Cells were transfected with a luminescent IFN-β-expressing TK Renilla-luciferase reporter plasmid (serving as an internal control for transfection efficiency normalization) and a Flag-tagged A104R plasmid in a dose-dependent manner. Poly (dA:dT) (InvivoGen) and 2′3′-cGAMP ligands (InvivoGen) were transfected with Lipofectamine 2000 (Invitrogen) and Lipofectamine RNAimax (Invitrogen), respectively, while other plasmids were transfected using PEI. QUANTI-Luci were utilized to measure the luciferase activity induced by poly (dA: dT), cGAS, or cGAMP on hSTING, with and without the presence of Flag-A104R. Luciferase activity was measured 24 h after transfection using the Dual-Luciferase Reporter Assay System (Promega; E1980) following the manufacturer’s guidelines.

### Nuclear fractionation assay

Cells were seeded in a six-well cell culture plate until they reached 80–90% confluency level. A104R protein stably expressing 3D4/21 cells were infected with ADV-GFP (MOI = 1.0) for 2 h, harvested at indicated timepoints. The nuclear and cytoplasmic fractions were extracted according to the manufacturer’s instructions (Invent Biotechnologies Inc., SM-005).

### Immunofluorescence and confocal analysis

PK-15 cells were seeded into an 8-well chamber slide (ibidi). 12 h later, cells were transfected with the indicated plasmids, and virus infection was done at 24 hpt, at the predetermined timepoints. Cells were then rinsed with PBS and fixed using 4% paraformaldehyde for 20 min. Followed by cells were permeabilized with methanol for 20 min at −20 °C. Cells were initially incubated with 2% BSA containing PBS for 1 h at room temperature. Subsequently, cells were incubated with specific primary antibodies for 12 h at 4 °C. After three PBST washes, cells were treated with secondary antibodies for 1 h at room temperature. Following another three washes using PBST, the nuclei were visualized by incubating with a mixture of diluted 4′,6-diamidino-2-phenylindole (DAPI) (Sigma) and 1 mg/mL RNase for 10 min. Images were taken using a Leica DMi8 microscope. The analysis was conducted using LAS-X software and ImageJ software.

### Mass spectrometry

HEK293T cells transfected with GST-vector or GST-A104R were harvested at 36 hpt. Cell pellets were collected using RIPA buffer, protease, and phosphatase inhibitor cocktail. The WCL was collected after sonication and centrifugation, followed by incubation with Sepharose 6B beads for 2 h at 4 °C. Precleared WCL was subjected to pull-down with glutathione Sepharose 4B beads overnight at 4 °C. The unbound proteins were washed away with lysis buffer and PBS. The proteins bound to the beads were eluted with SDS sample buffer, and separated by 4–15% NuPAGE gels (Invitrogen; NP0323PK2), followed by silver staining. Protein bands present exclusively in the gel were subjected to mass spectrometry.

### Molecular docking

Interactions between A104R (GenBank: FR682468.1) and KPNA3 (Uniprot ID: A0A5G2R9E6_PIG), or A104R and KPNA6 (Uniprot ID: A0A4X1VYA1_PIG) were predicted by AlphaFold3 server [25] and visualized in ChimeraX 1.9 [26]. The interacting residues were identified by PYTHON pipeline [27], keeping a 3.0 or 3.5 Å interatomic distance cutoffs.

### Statistical analysis

Statistical analyses were performed using GraphPad Prism 8.0. Data are presented as the mean ± standard deviation (SD) of three biological replicates from a representative experiment. Statistical significance between two independent groups were compared by an unpaired Student’s *t*-test. Statistical significance among groups was determined by one-way analysis of variance (ANOVA) followed by Tukey’s test for multiple comparisons. Statistical significance was indicated as *P ≤ 0.05, **P ≤ 0.01, *** *P* ≤ 0.001, or **** *P* ≤ 0.0001. ns: not significant.

## Results

### A104R inhibits IFN-β promoter activation downstream of IRF3

Viruses employ an array of techniques that enhance their replication within the host and effectively inhibit type I IFN signaling. To examine the potential role of A104R in altering the type I IFN signaling, we conducted separate screening on 158 viral proteins originating from ASFV by using an ADV-GFP reporter gene assay system as mentioned our previous study [24]. Distinct ASFV viral genes or a control vector were transiently transfected into PK-15 cells. At 24 hpt, cells were infected with ADV-GFP at an MOI = 1.0. At 24 hpi, cells harboring the virus were harvested, and virus replication was measured. Through this screening, we identified several ASFV proteins that facilitate enhanced viral replication. Among them, we identified that the quantity of cells expressing GFP was significantly greater in those cells expressing A104R compared with the control cells (Additional file 1). This pronounced effect prompted us to select A104R for subsequent detailed molecular characterization of its immune evasion mechanisms.

**Figure 1:**
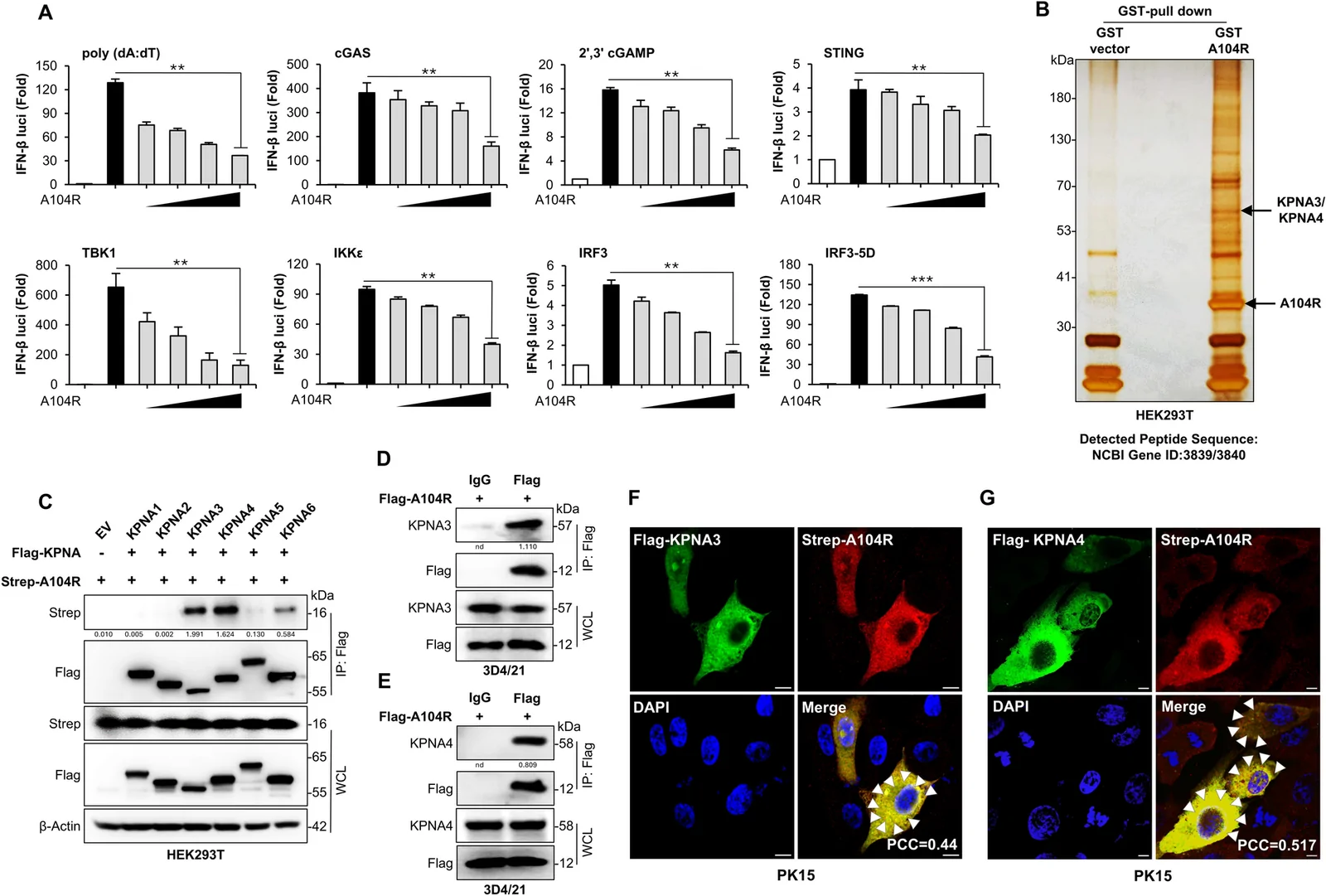
**A104R targets of KPNA proteins and inhibits type I IFN signaling**. **A** HEK293T cells were cotransfected with an IFN-β promoter-driven firefly luciferase reporter, TK-Renilla control, the indicated stimulators (STING, TBK1, IKKε, IRF3, or IRF3-5D), and increasing amounts of Flag-A104R. For poly(dA:dT) stimulation, cells received both reporters, Flag-A104R, and poly(dA:dT). Firefly activity was normalized to Renilla activity at 24 h. For cGAS- and cGAMP-induced assays, 293-Dual hSTING-A162 cells expressing Flag-A104R were transfected with 3×Flag-cGAS or stimulated with 2′3′-cGAMP. Secreted IFN-β-dependent Lucia luciferase activity was measured after 12 h. White, black, and gray bars represent negative control, positive control, and increasing Flag-A104R doses, respectively. **B** Silver-stained GST-A104R pull-down products analyzed by mass spectrometry. The positions of GST-A104R, KPNA3 (UniProt O00505), and KPNA4 (UniProt O00629) are indicated. **C** HEK293T cells were cotransfected with Flag-KPNA1–6 or control vector and Strep-A104R. After 36 h, anti-Flag immunoprecipitates were immunoblotted with the indicated antibodies. Values show Strep-A104R intensity normalized to the corresponding Flag bait. **D**, **E** Flag-A104R-expressing or control 3D4/21 cells were subjected to anti-Flag immunoprecipitation and immunoblotting for KPNA3 (**D**) or KPNA4 (**E**). Values indicate KPNA3 or KPNA4 intensity normalized to immunoprecipitated Flag-A104R. **F**, **G** Confocal microscopy of PK-15 cells co-expressing Strep-A104R and Flag-KPNA3 (**F**) or Flag-KPNA4 (**G**). A104R, KPNA proteins, and nuclei are shown in red, green, and blue, respectively; yellow indicates colocalization. Scale bar indicates 20 μm. Data are representative of three independent experiments. Values represent the mean ± SD of three biological replicates from a representative experiment (**A**). Unpaired Student’s *t*-test: *P ≤ 0.05, **P ≤ 0.01, *** *P* ≤ 0.001, or **** *P* ≤ 0.0001.

Upon viral infection, host sensor molecules activate type I IFN signaling cascades. We performed a luciferase promoter assay to determine the precise target of the cGAS-STING signaling cascades controlled by A104R. This assay entailed simultaneously expressing the A104R gene alongside cGAS-STING pathway-associated adaptor molecules to thoroughly investigate the impact of A104R on the activation of the virus-induced type I IFN signaling cascade. Here, we found that A104R markedly suppressed the activation of the IFN-β promoter activity induced by poly (dA:dT), cGAS, 2',3'-cGAMP, STING, TBK1, IKKϵ, IRF3, and IRF3-5D (a constitutively active form of IRF3) in a dose-dependent manner (Figure 1A). These findings indicate that A104R exerts regulatory control over the type I IFN signaling pathway at a level or beneath that of IRF3. To investigate the possibility of interaction between A104R and IRF3 occurring at endogenous protein levels, WCL and immunoprecipitants obtained with Flag antibody or mouse preimmune serum (IgG) were subjected to immunoblotting with anti-IRF3 and anti-Flag antibodies. Data revealed that A104R did not interact with endogenous IRF3 (Additional file 2A). Based on the data, it indicates that the inhibitory effect of A104R on type I IFN signaling was not caused by direct interaction with IRF3.

### A104R targets KPNA to suppress the type I IFN pathway

In our pursuit of identifying a precise target molecule of A104R in the context of innate immune responses, we introduced GST-tagged A104R into HEK293T cells and performed a large-scale GST pull-down assay (Figure 1B). Mass spectrometry analysis identified KPNA3 (UniProt: O00505) and KPNA4 (UniProt: O00629) as interacting proteins. These findings led us to hypothesize that A104R interacts with KPNA to inhibit type I IFN signaling. To assess the potential interaction between A104R and the nuclear localization signal receptor, KPNA, we carried out an IP assay. Flag-tagged KPNA 1, 2, 3, 4, 5, and 6 expression plasmids were transfected with the empty vector or Strep-tagged A104R. At 36 hpt, immunoprecipitations were performed with an anti-Flag antibody, and the precipitated proteins were analyzed by immunoblotting with antibodies recognizing Strep-tagged A104R. Interestingly, A104R exclusively coprecipitated with KPNA 3, 4, and 6 (Figure 1C). Subsequently, we confirmed the A104R interaction with KPNA3 and KPNA4 by IP assays in A104R stably expressing 3D4/21 cells (Figure 1D and E). Furthermore, HEK293T cells were transfected with a Flag-tagged or Strep-tagged A104R plasmid, 36 hpt, and IP assays were carried out with an anti-Flag or anti-Strep antibody. The precipitated proteins were analyzed by immunoblotting. KPNA3 (Additional file 2B) and KPNA4 (Additional file 2C) coprecipitated with A104R. Structural modeling further confirms the interaction between A104R and KPNA3. The 3D surface-rendered model indicates a stable binding configuration between the N-termini of both A104R and KPNA3 (Additional file 2D). The ribbon diagram was used to identify the interacting residues. The N-terminal residues; KPNA3-ASP92, GLY135, THR140, TRP169, ASN173, ASP177, ASN209, ASN216, ARG219, ARG219, ARG219, and ASP251-interacted with the N-terminal residues; THR10, LYS4, LYS4, LYS5, LYS5, LYS4, LYS4, THR3, MET1, MET1, THR3, and MET1 of A104R, respectively (Additional file 2E). Further, after transfection of PK-15 cells, colocalization of A104R with KPNA3 and KPNA4 was observed using confocal microscopy (Figure 1F, G). Based on these results, we confirmed that A104R can indeed interact with KPNA3 and KPNA4.

**Figure 2:**
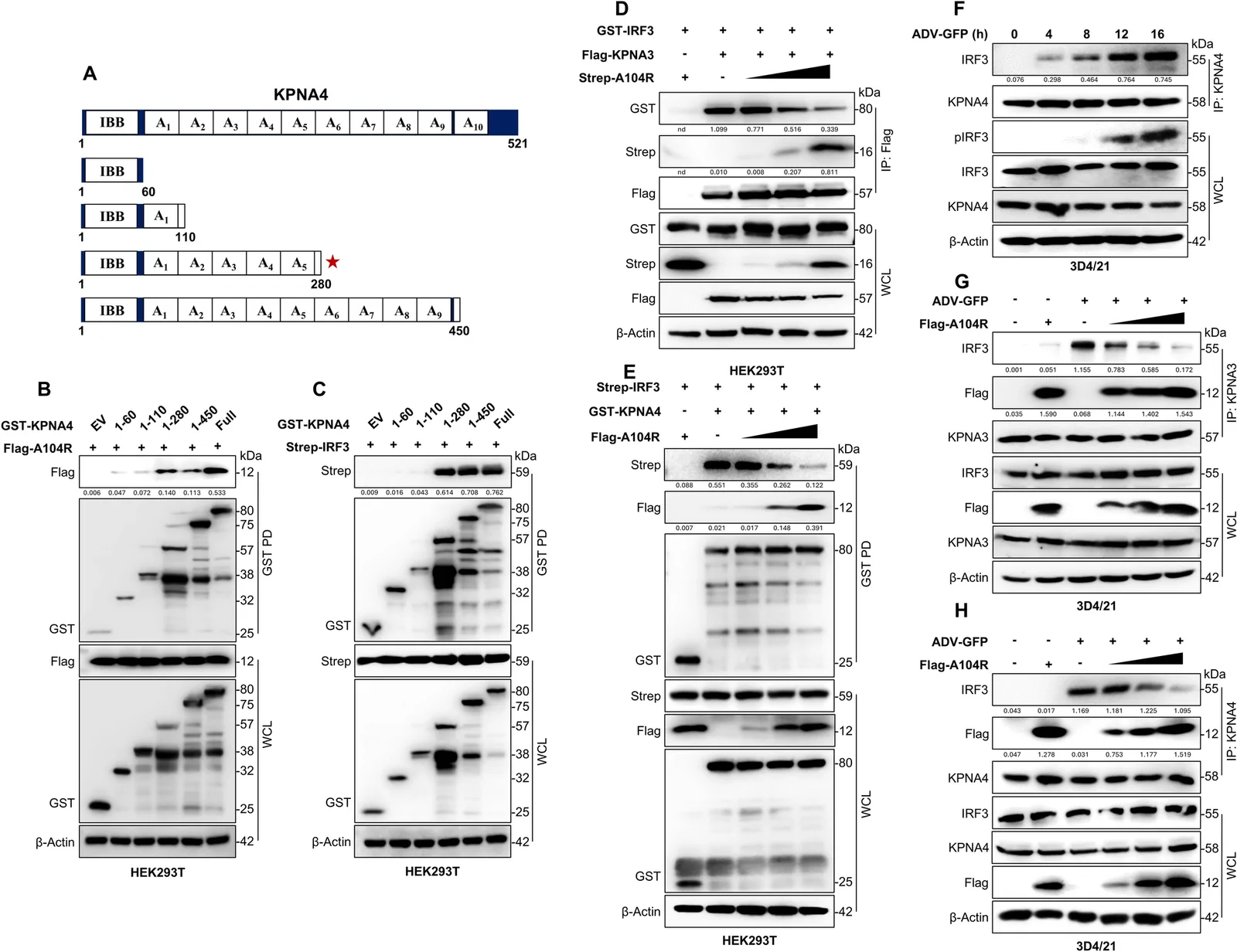
**A104R targets KPNA proteins and disrupts the interaction of IRF3 with KPNA3 and KPNA4.** **A** Schematic representation of the GST-tagged KPNA4 constructs. Predicted molecular weights were 25 kDa for GST-vector; 32, 38, 57, and 75 kDa for GST-KPNA4 residues 1–60, 1–110, 1–280, and 1–450, respectively; and 80 kDa for full-length KPNA4 (521 aa). **B**, **C** HEK293T cells were cotransfected with the indicated GST-KPNA4 constructs and Flag-A104R (**B**) or Strep-IRF3 (**C**). Cell lysates were subjected to GST pull-down and immunoblotting with the indicated antibodies. Values represent Flag-A104R (**B**) or Strep-IRF3 (**C**) intensity normalized to the corresponding GST bait. **D** HEK293T cells were cotransfected with Flag-KPNA3, GST-IRF3, and Strep-A104R. After 36 h, anti-Flag immunoprecipitates were analyzed by immunoblotting. GST-IRF3 and Strep-A104R intensities were normalized to immunoprecipitated Flag-KPNA3. **E** HEK293T cells were cotransfected with GST-KPNA4, Strep-IRF3, and Flag-A104R. After 36 h, GST pull-down products were analyzed by immunoblotting. Strep-IRF3 and Flag-A104R intensities were normalized to pulled-down GST-KPNA4. **F** 3D4/21 cells were infected with ADV-GFP for the indicated periods. KPNA4 immunoprecipitates were analyzed for associated IRF3, and IRF3 intensity was normalized to immunoprecipitated KPNA4. **G**, **H** 3D4/21 cells were transfected with increasing amounts of Flag-A104R or control vector and subsequently infected with ADV-GFP. Cell lysates were subjected to KPNA3 (**G**) or KPNA4 (**H**) immunoprecipitation and immunoblotting. IRF3 and Flag-A104R intensities were normalized to the corresponding immunoprecipitated KPNA3 (**G**) or KPNA4 (**H**).

Thus far, the findings have consistently demonstrated the interaction between A104R and KPNA3 and KPNA4. To pinpoint the specific domain within KPNA that engages with A104R, we generated four GST-tagged KPNA4 fragments. (1-60aa, 1-110aa, 1-280aa, and 1-450aa) (Figure 2A). In a GST pull-down assay involving each construct along with A104R, it was evident that A104R exhibited an interaction with KPNA4 within the region defined by aa 110–280, which harbors a nuclear localization signal essential for recognizing cargo molecules (Figure 2B). Large macromolecules (>40 kDa) are transported through the nucleus and the cytoplasm via nuclear pore complexes (NPCs) using signal-dependent, carrier-mediated mechanisms. Cargo proteins containing classical NLSs are generally recognized by importin-α in conjunction with importin-β. The cargo-importing complex translocates to the nucleus via transient interaction with the phenylalanine-glycine repeat region of nucleoporins, which are components of NPCs [23]. It has been reported that nuclear import of both IRF3 is mediated by KPNA3 and KPNA4 [28, 29]. Therefore, it was of interest to determine if A104R binds to a region similar to the one where KPNA and IRF3 bind. The same four KPNA4 expression constructs were investigated for IRF3 interaction. Strep-tagged IRF3 was transfected alongside the KPNA4 domains at 36 hpt, and a GST pull-down assay was conducted. The precipitated material was subjected to immunoblotting. Intriguingly, our findings revealed that IRF3 shares an interaction region with KPNA4 that aligns with where A104R interacts with KPNA4 (Figure 2C). Next, we examined the direct competition paradigm by evaluating the ability of A104R to suppress KPNA3 and KPNA4 interaction with IRF3. To determine if there is a dose-dependent effect of A104R on the interaction between KPNA3 and KPNA4 with IRF3, we co-expressed GST-IRF3 with Flag-KPNA3 (Figure 2D) and Strep-IRF3 with GST-KPNA4 (Figure 2E) with or without KPNA and A104R, either was not expressed or was expressed in increasing amounts in HEK293T cells, followed by IP with anti-Flag antibody or with GST bead accordingly. The results suggest that the increase in the interaction between A104R and KPNA was accompanied by a decrease in the interaction between KPNA and IRF3.

Next, we delved into the interactions between IRF3, and KPNA molecules following viral infection and determined the optimal timepoint for the association between cellular IRF3 and KPNA. The 3D4/21 cells were infected with ADV-GFP (MOI = 1.0) at different time intervals. The interaction between IRF3 and KPNA4 under endogenous conditions was then observed through IP analysis. As depicted in Figure 2F, the association between IRF3 and KPNA molecules progressively intensified, reaching its maximum level 16 hpi. Then we tested whether the interactions of IRF3 with KPNA3 and KPNA4 were affected by the expression of A104R under endogenous conditions. The 3D4/21 cells were transfected with increasing amounts of the Flag-A104R plasmid, followed by ADV-GFP infection. Endogenous IRF3 co-immunoprecipitated with the KPNA, suggesting that smaller amounts of IRF3 interacted with KPNA3 and KPNA4 in cells expressing larger amounts of A104R (Figures. 2G, H). Conversely, an overexpression recovery assay was conducted to confirm the competitive interaction between A104R and IRF3 for KPNA3 or KPNA4. The 3D4/21 cells were transfected with increasing amounts of Flag-tagged KPNA3 or GST-tagged KPNA4 plasmids, followed by ADV-GFP infection. Endogenous IRF3 profoundly immunoprecipitated with KPNA3 (Additional file 3A) and KPNA4 (Additional file 3B), while interacting with A104R too. These data confirm that A104R competitively inhibited the interaction of IRF3 with KPNA3 and KPNA4.

### A104R does not inhibit phosphorylation and dimerization of IRF3, but does inhibit the nuclear translocation upon virus infection

**Figure 3:**
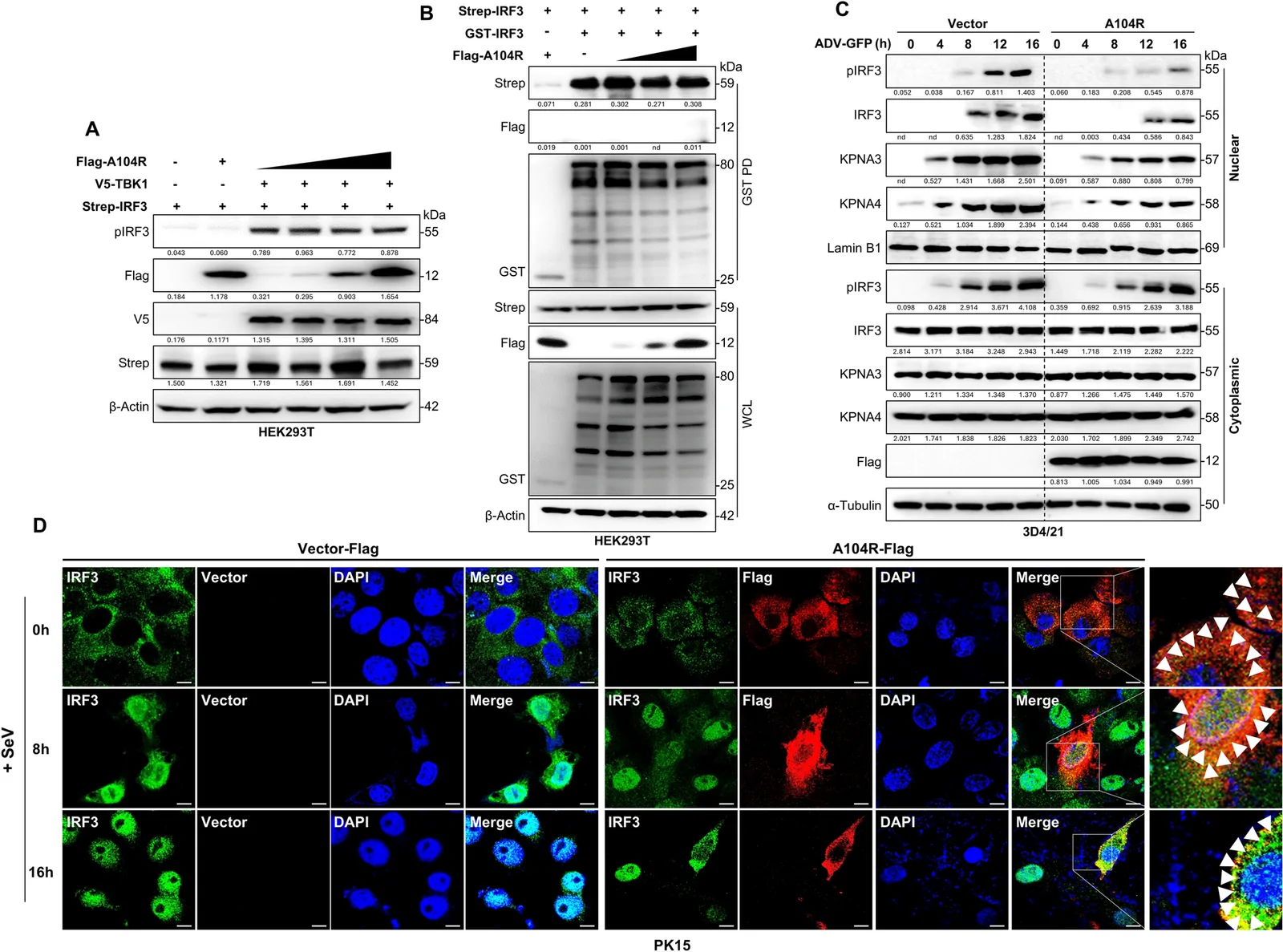
**A104R inhibits nuclear translocation without affecting IRF3 phosphorylation or dimerization.** **A** HEK293T cells were cotransfected with V5-tagged TBK1, Strep-tagged IRF3, and increasing amounts of Flag-tagged A104R. At 36 hpt, cell lysates were subjected to immunoblotting with the indicated antibodies. The numerical values below the panels represent the relative intensity normalized to the β-actin (internal control). **B** HEK293T cells were cotransfected with Strep-tagged IRF3, GST-tagged IRF3, and increasing amounts of Flag-tagged A104R, balanced with the control vector. At 36 hpt, cell lysates were subjected to GST pull-down and immunoblotting. The numerical values below the anti-Strep and -Flag panels in the GST PD represent the relative intensity of the Strep-IRF3 and Flag-A104R signals, normalized to the corresponding GST-IRF3 bait in the same GST PD. **C** Stable 3D4/21 cells expressing Flag-A104R were infected with ADV-GFP at the indicated timepoints. Cytoplasmic and nuclear fractions were prepared using a Nuclear and Cytoplasmic Protein Extraction Kit and analyzed separately by immunoblotting with indicated antibodies. The numerical values below the panels in the nuclear and cytoplasmic fractions represent the relative intensity normalized to the internal controls of Lamin B1 and α-Tubulin, respectively. **D** Control- and Flag-A104R-expressing PK-15 cells were infected with Sendai Virus. At the indicated timepoints, A104R was detected with anti-Flag antibody and a Cy3-conjugated secondary antibody. IRF3 and A104R were shown in green and red, respectively. Nuclei were stained with DAPI (blue). Yellow indicates colocalization and is marked with arrows in the enlarged images. Data are representative of three independent experiments. Scale bar indicates 20 μm.

IRF3 is a key transcription factor in IFN-mediated signalling. IRF3 is activated after phosphorylation by the kinases IKKϵ and TBK1 [30]. To investigate the influence of A104R on IRF3 activation, we first assessed IRF3 phosphorylation in the presence of A104R. We induced IRF3 phosphorylation by overexpressing TBK1, and vector plasmid or A104R was introduced in a dose-dependent manner into HEK293T cells. Our observations suggest that escalating levels of A104R overexpression had no effect on IRF3 phosphorylation (Figure 3A). Subsequently, we examined the inhibitory effect of A104R on IRF3 dimerization through a competition assay by co-expressing GST-IRF3 and Strep-IRF3 proteins in HEK293T cells, along with increasing amounts of Flag-A104R plasmid. In the results obtained from a GST pull-down assay, it was evident that the levels of GST-IRF3 and Strep-IRF3 homodimers remained unaffected as A104R expression increased (Figure 3B). In contrast, it is important to highlight that we were unable to detect any interaction between GST-IRF3 and Flag-A104R in the context of this experiment. Nuclear translocation of phosphorylated dimerized IRF3 is critical for initiating IFN-β transcription. Since A104R does not affect IRF3 phosphorylation or dimerization, we then investigated A104R effect on IRF3 nuclear translocation. The 3D4/21 cells stably harboring control vector and A104R were subjected to ADV-GFP infection at indicated timepoints. Subsequently, cytoplasmic and nuclear fractions were isolated. The levels of phosphorylated IRF3, total IRF3, KPNA3, and KPNA4 were then assessed via immunoblotting (Figure 3C). Phosphorylation of IRF3 was observed in both cytoplasm and nuclear fraction at 8 hpi, and the level increased up to 16 hpi. The amounts of phosphorylated IRF3 in the cytoplasm of control and A104R overexpressed cells also increased with time, whereas the amount of phosphorylated IRF3 in the cytoplasmic fraction from A104R expressing cells did not differ from that of control cells. Results further demonstrate that A104R did not affect the levels of IRF3, KPNA3, and KPNA4 in cytoplasm. However, A104R expression significantly reduced the expression of IRF3, KPNA3, and KPNA4 in the nucleus after virus infection with time. To further confirm the obtained results, we finally analyzed the IRF3 nuclear localization inhibition via confocal microscopy. It showed that IRF3 was distributed in the cytoplasm with or without overexpression of A104R in uninfected cells. In cells transfected with control vectors, IRF3 was efficiently translocated to the nucleus upon Sendai virus (SeV) infection. However, SeV-induced IRF3 nuclear translocation was impaired in cells expressing A104R (Figure 3D). The results suggest that A104R impairs the nuclear import of IRF3 following viral infection.

### A104R inhibits the transcription of antiviral genes and cGAS-STING pathway signaling

**Figure 4:**
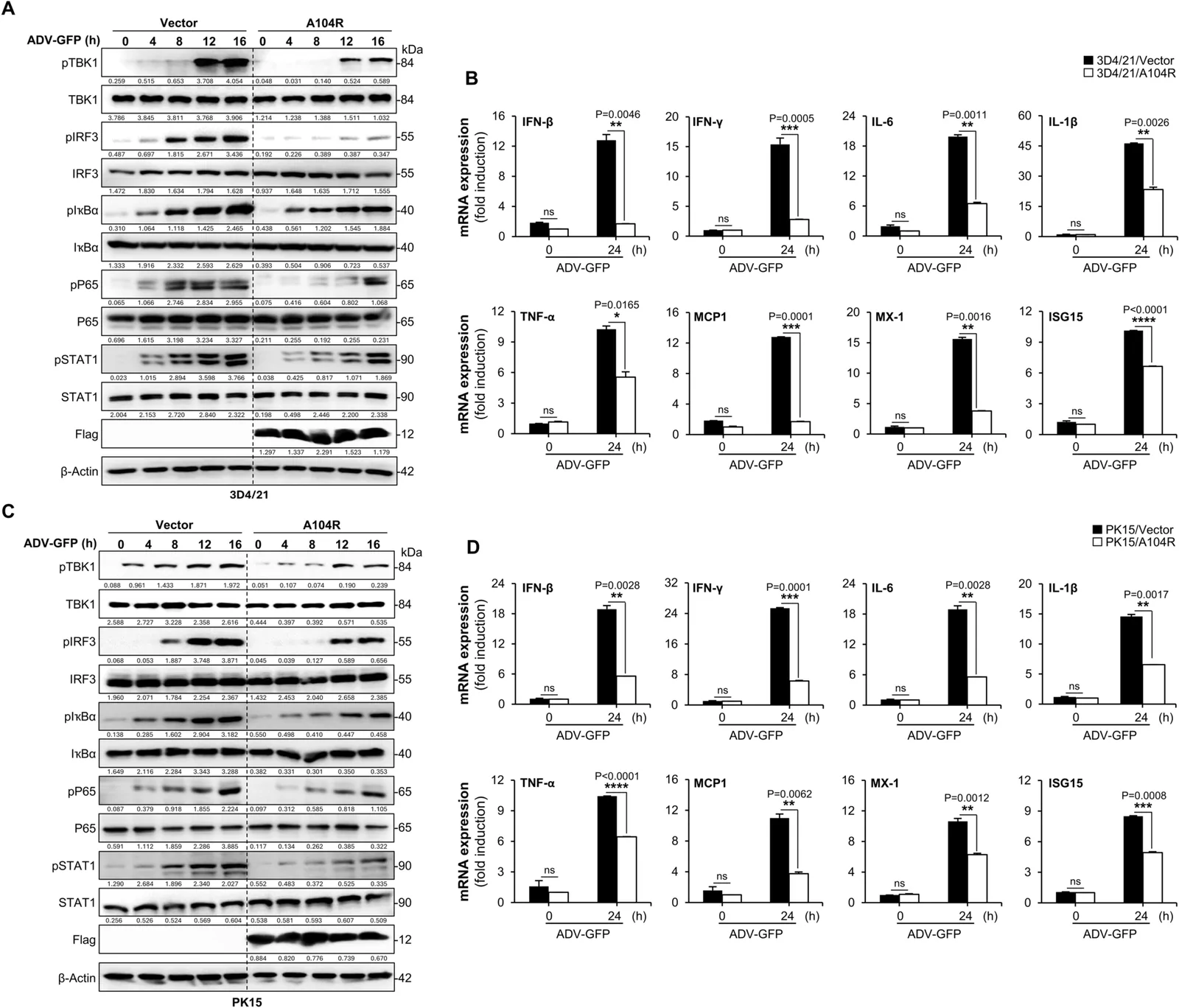
**A104R inhibits antiviral gene transcription and cGAS-STING pathway signaling.** **A** The 3D4/21 cells were transfected with Flag-tagged A104R or the control vector and infected with ADV-GFP at a MOI of 1. At the indicated timepoints, A104R expression and the levels of total and phosphorylated TBK1, IRF3, IκBα, p65, and STAT1 were detected by immunoblotting. The numerical values below the panels represent the relative intensity normalized to the β-actin (internal control). **B** The 3D4/21 cells were transfected with Flag-tagged A104R or the Flag control vector. At 24 hpt, cells were infected with ADV-GFP at a multiplicity of infection (MOI) of 1.0. At the indicated timepoints, total RNA was extracted, and the mRNA levels of the indicated genes were measured by RT-qPCR. The numerical values below the anti-Strep and -Flag panels in the GST PD represent the relative intensity of the Strep-IRF3 and Flag-A104R signals, normalized to the corresponding GST-IRF3 bait in the same GST PD. **C** PK-15 cells were transfected with Flag-tagged A104R or the control vector and infected with ADV-GFP at an MOI of 1.0 At the indicated timepoints, A104R expression and the levels of total and phosphorylated TBK1, IRF3, IκBα, p65, and STAT1 were analyzed by immunoblotting. The numerical values below the panels represent the relative intensity normalized to the β-actin (internal control). **D** PK-15 cells were transfected with Flag-tagged A104R or the control vector and infected with ADV-GFP at an MOI of 1.0 at 24 hpt. At the indicated timepoints, total RNA was extracted, and the mRNA levels of the indicated genes were measured by RT-qPCR. Data are representative of three independent experiments. Values represent the mean ± SD of three biological replicates from a representative experiment. Unpaired Student’s *t*-test: *P ≤ 0.05, **P ≤ 0.01, *** *P* ≤ 0.001, or ****P ≤ 0.0001. ns: not significant.

To further validate A104R’s effects on virus-induced type I IFN signaling, we investigated DNA virus-mediated phosphorylation of TBK1, IRF3, inhibitor of nuclear factor kappa B, alpha (IκBα), RELA proto-oncogene (p65), and STAT1, all of which are key signaling molecules within the type I IFN pathway in 3D4/21 cells (Figure 4A) and PK-15 cells (Figure 4C). In this experiment, A104R-overexpressing 3D4/21 and PK-15 cells and the corresponding control cells were infected with ADV-GFP and harvested 0, 4, 8, 12, and 16 h later for immunoblot analysis. Phosphorylation of cGAS-STING signaling molecules and Nuclear factor kappa-light-chain-enhancer of activated B cells (NF-κB)-related signaling molecules was assessed at the indicated timepoints after infection. Phosphorylation levels of TBK1, IRF3, IκBα, P65, and STAT1 were notably reduced in A104R-expressing 3D4/21 cells and PK-15 cells compared with the control cells. These findings provide compelling evidence in favor of the hypothesis that A104R plays a role in inhibiting type I IFN signaling pathway. To further confirm the effects of A104R on antiviral signaling cascades, we investigated DNA virus-mediated transcription of IFNs (IFN-β and IFN‑gamma [IFN-γ]), pro-inflammatory cytokines (IL-6, IL-1β, and tumor necrosis factor alpha [TNF-α]), and IFN-inducible genes (MCP-1, MX-1, ISG-15, PKR, IFIT1, IFITM3, OASL) in A104R-overexpressed 3D4/21 stable cell line and A104R-overexpressed PK-15. For this experiment, we infected cells with ADV-GFP and performed RT-qPCR using specific primers (Table 2). Consequently, in cells expressing A104R, the mRNA expression levels of IFNs and other antiviral genes (Figures 4B, D, and Additional files 4A, B) were notably reduced compared with those in control cells. Taken together, these findings indicate that the interaction of A104R with KPNA3 and KPNA4 significantly impairs host type I IFN signaling pathways, consequently suppressing the transcription of antiviral genes.

### ASFV A104R negatively regulates antiviral immune responses against viral infection

**Figure 5:**
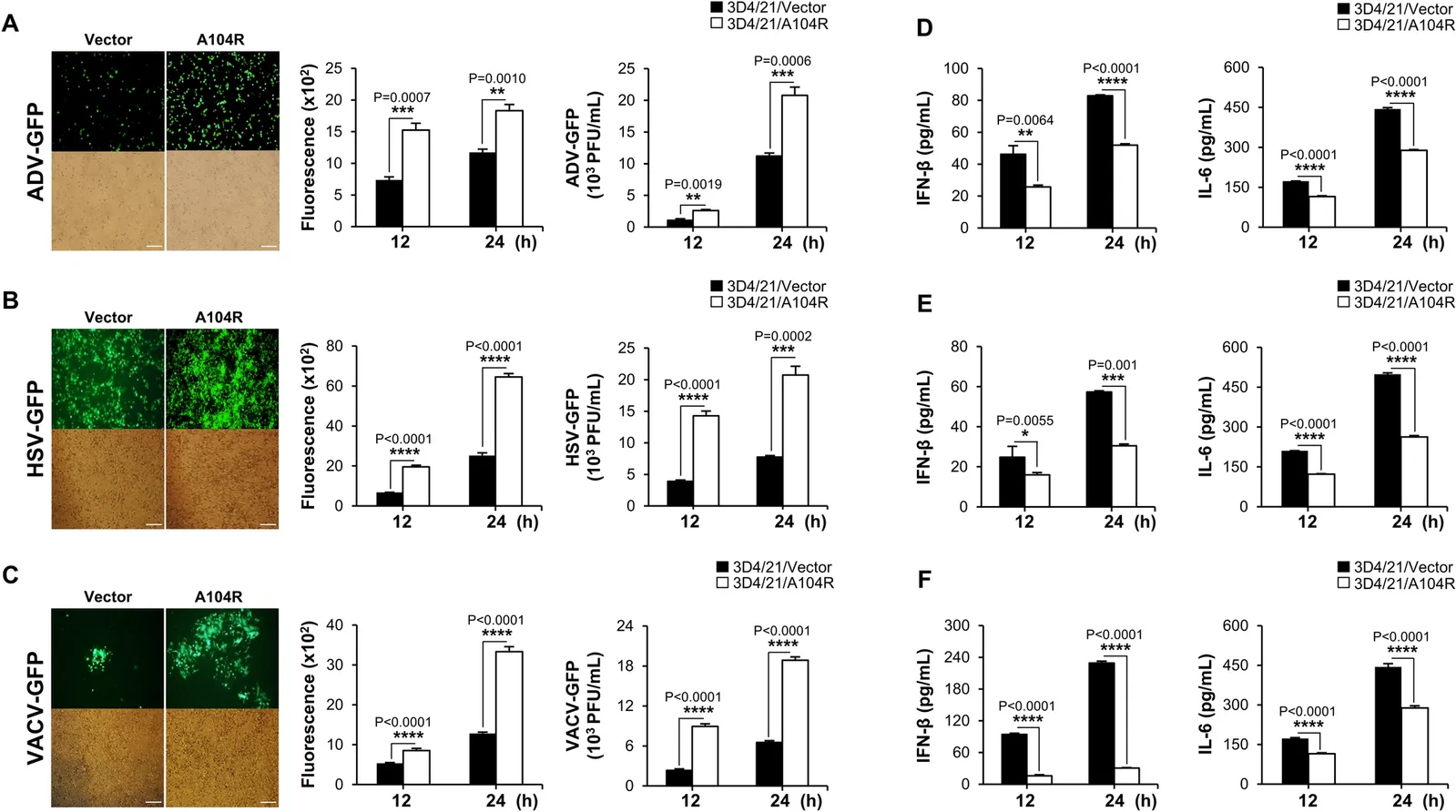
**A104R negatively regulates antiviral immune responses in stable 3D4/21 cells.** **A–C **Stable 3D4/21 cells expressing Flag-A104R or the control vector were infected with ADV-GFP, HSV-GFP, or VACV-GFP, respectively, at a multiplicity of infection (MOI) of 1.0. At 12 and 24 h post-infection (hpi), fluorescence intensity levels were quantified, and GFP fluorescence images were captured at 24 hpi. Standard plaque assays were performed in A549 or Vero cells for viral titer determination. **D–F** Porcine IFN-β and IL-6 concentrations in culture supernatants collected at 12 and 24 hpi were measured by ELISA. Data are representative of three independent experiments. Values represent the mean ± SD of three biological replicates from a representative experiment. Unpaired Student’s *t*-test: *P ≤ 0.05, **P ≤ 0.01, *** *P* ≤ 0.001, or **** *P* ≤ 0.0001. Scale bar indicates 50 μm.

To provide additional evidence about the crucial function of A104R on antiviral immune response and virus replication in vitro, we used 3D4/21 and MA-104 cells stably expressing Flag-tagged A104R or a control vector. Additionally, we transfected PK-15 cells with a Flag-tagged A104R or a control vector. Each cell experienced infections from substitute viruses, ADV-GFP, HSV-GFP, or VACV-GFP, an inevitable replacement for ASFV. All viruses are known to be IFN-sensitive, as ASFV. The efficiency of the transient transfection of plasmids in each cell line was confirmed by immunoblotting or RT-qPCR (Additional files 5A–F). Notably, we found that virus replication in A104R overexpressed cells was significantly higher than that in control cells in the three cell lines (Figure 5A, B, C and Additional files 6A–D). Using ELISA, we further determined the levels of IFN-β and IL-6 secreted by each virus-infected cell (Figure 5D, E, F and Additional files 5E, H). Gene overexpression cells released fewer cytokines than control cells, consistent with viral replication results. Together, these data suggested that A104R inhibits the production of type I IFNs and pro-inflammatory cytokines while increasing DNA virus replication in macrophages and epithelial cells.

### A104R and phosphorylated STAT1 bind to overlapping regions of KPNA6

**Figure 6:**
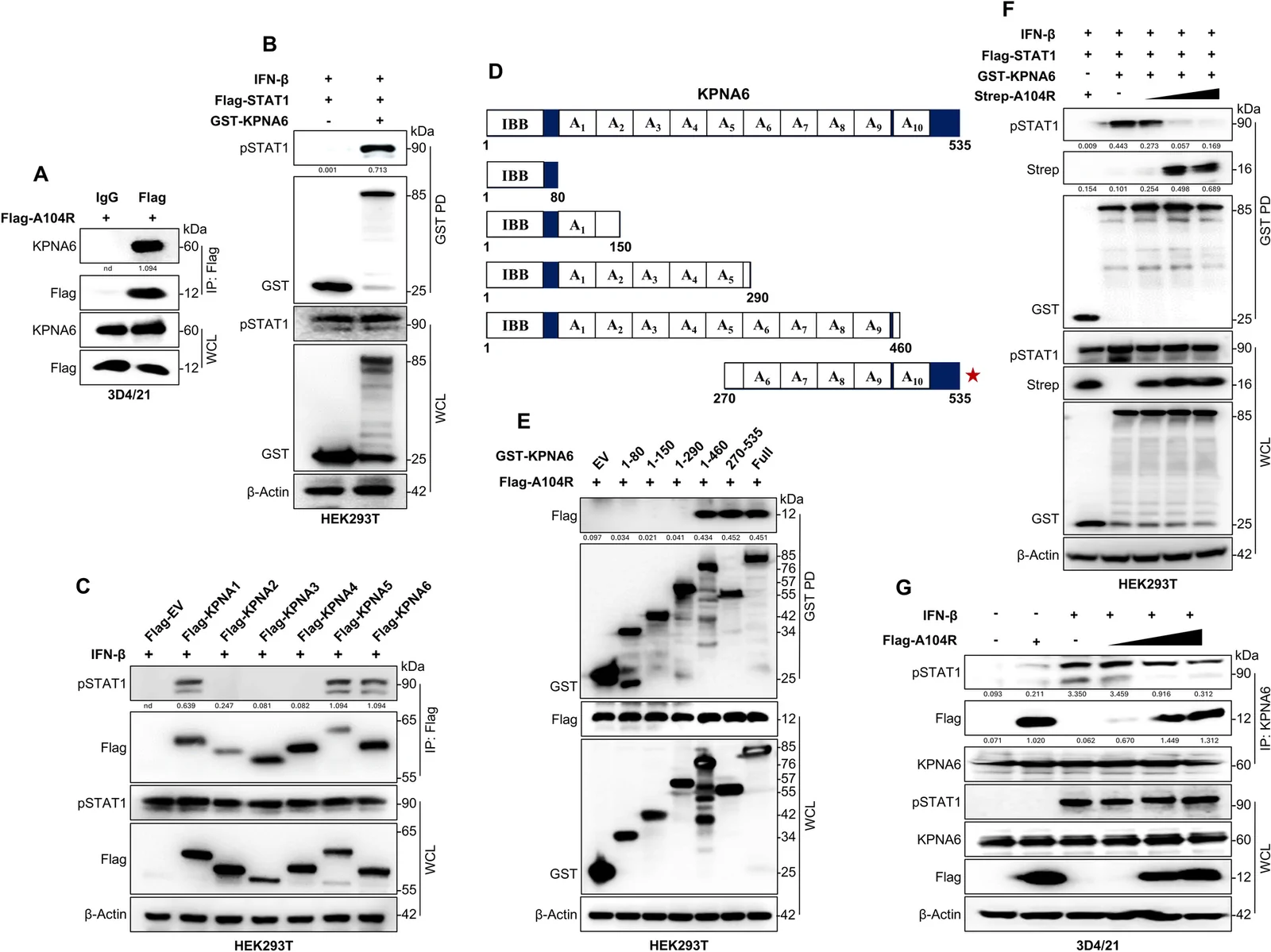
**A104R and phosphorylated STAT1 bind to overlapping regions of KPNA6.** **A** Flag-A104R-expressing stable 3D4/21 cells were subjected to anti-Flag immunoprecipitation and immunoblotting. KPNA6 intensity was normalized to immunoprecipitated Flag-A104R. **B** HEK293T cells co-expressing Flag-STAT1 and GST-KPNA6 were treated with IFN-β (1000 U/mL) for 1 h at 36 hpt. GST pull-down products were analyzed by immunoblotting, and phosphorylated STAT1 (pSTAT1) intensity was normalized to pulled-down GST-KPNA6. **C** HEK293T cells expressing Flag-KPNA1–6 were treated with IFN-β (1000 U/mL) for 1 h and subjected to anti-Flag immunoprecipitation. pSTAT1 intensity was normalized to the corresponding immunoprecipitated Flag-KPNA bait. **D** Schematic representation of GST-tagged KPNA6 constructs. Predicted molecular weights were 34, 42, 55, and 76 kDa for residues 1–80, 1–150, 1–290, and 1–460, respectively; 55 kDa for residues 270–535; and 85 kDa for full-length KPNA6 (535 aa). **E** HEK293T cells co-expressing GST-KPNA6 constructs and Flag-A104R were subjected to GST pull-down and immunoblotting. Flag-A104R intensity was normalized to the corresponding GST bait. **F** HEK293T cells co-expressing GST-KPNA6, Flag-STAT1, and Strep-A104R were harvested at 36 h for GST pull-down and immunoblotting. pSTAT1 and Strep-A104R intensities were normalized to GST-KPNA6. **G** Flag-A104R-expressing 3D4/21 cells were treated with IFN-β (1000 U/mL) for 1 h and subjected to KPNA6 immunoprecipitation and immunoblotting. pSTAT1 and Flag-A104R intensities were normalized to immunoprecipitated KPNA6.

In addition to KPNA3 and KPNA4, A104R was found to interact with KPNA6 (Figure 1C). To confirm if A104R is also able to interact with endogenous KPNA6, 3D4/21 cells were transfected with a Flag-tagged A104R, and IP was conducted using an anti-Flag antibody, and the resulting precipitate was examined by immunoblotting. Upholding our results, KPNA6 was found to coprecipitate with A104R (Figure 6A). This result confirms that A104R is indeed able to interact with KPNA6. Reports indicate that the NPI-1 subfamily of KPNA (KPNA1, KPNA5, and KPNA6) interacts with PY-STAT1 to facilitate the nuclear translocation of ISGF3 [31, 32]. Our subsequent inquiry was centered on the interplay between KPNA6 and PY-STAT1. HEK293T cells transfected with GST-tagged KPNA6 and Flag-tagged STAT1 and Human IFN-β (1000 U/mL) were treated with cells for 1 h and underwent a GST pull-down assay. Data revealed that KPNA6 indeed participates in interaction with PY-STAT1 (Figure 6B). To further confirm the interaction between PY-STAT1 and with NPI-1 subfamily of, KPNA, HEK293T cells were transfected with Flag-tagged KPNA1, 2, 3, 4, 5, and 6 expression plasmids or a control vector, and cells were stimulated with (1000 U/mL) IFN-β for 1 h, and iIP was performed. Immunoblot analysis revealed that PY-STAT1 coprecipitated exclusively with Flag-KPNA1, KPNA5, and KPNA6 (Figure 6C), consistent with findings from previous studies [31, 32]. We further assessed A104R-KPNA6 interaction using AlphaFold3-based structural modeling combined with PYTHON pipeline.

The 3D surface-rendered model indicates an interaction between A104R with the C-terminal KPNA6 (Additional file 7A). Further the ribbon diagram was generated to identify the interacting residues. The C-terminal residues of KPNA3-GLU471, ASP477, GLU480, TYR491, and PHE495-interacted with the C-terminal residues ARG69, LYS85, HIS88, ILE93, and ILE93 of A104R, respectively (Additional file 7B). Together, these structural predictions suggest that A104R directly associates with KPNA6 through a specific interface that may influence KPNA6-mediated nuclear transport.

For nucleus trafficking, PY-STAT1 interacts with KPNA1 and KPNA5 C-terminal Armadillo repeats (ARM repeats) [31, 33]. Within the NPI-1 subfamily, KPNA1 and KPNA5 share greater than 80% sequence similarity with KPNA6 [34]. This prompted our curiosity about whether A104R binds to a similar region of the KPNA6. As depicted in Figure 6D, we generated five distinct GST-tagged KPNA6 expression domains (covering amino acid sequences 1–80, 1–150, 1–290, 1–460, and 270–535). In a GST pull-down assay involving each of these constructs along with Flag-tagged A104R, a robust binding affinity was observed with the C-terminal 6–10 ARM repeats region. Interestingly, this includes the same region where PY-STAT1 interacts with the NPI-1 subfamily of KPNA. As illustrated in Figure 6E. Using the direct competition paradigm, we tested whether A104R could prevent PY-STAT1 from interacting with KPNA6. HEK293T cells were transfected with Flag-STAT1, GST-KPNA6, or Strep-A104R. At 36 hpt, the cells were treated with IFN-β for 1 h, a GST pull-down assay was then performed, and the immunoprecipitated material was subjected to immunoblotting. As shown in Figure 6F, the A104R protein exhibited dose-dependent inhibitory activity toward the interaction between PY-STAT1 and KPNA6. We further validated the results obtained using 3D4/21 cells in the endogenous competition model. PY-STAT1 coprecipitated with KPNA6 in the absence and presence of low concentrations of A104R. However, as the A104R concentration increased, the ability of KPNA6 to coprecipitate with PY-STAT1 was inhibited, whereas A104R itself coprecipitated with KPNA6 (Figure 6G). This evidence of direct competition supports a model in which A104R inhibits the nuclear localization of PY-STAT1 by competing with KPNA6.

### A104R binding to KPNA6 does not alter STAT1 phosphorylation and ISGF3 formation but shows diminished inhibition of STAT1 nuclear transport

**Figure 7:**
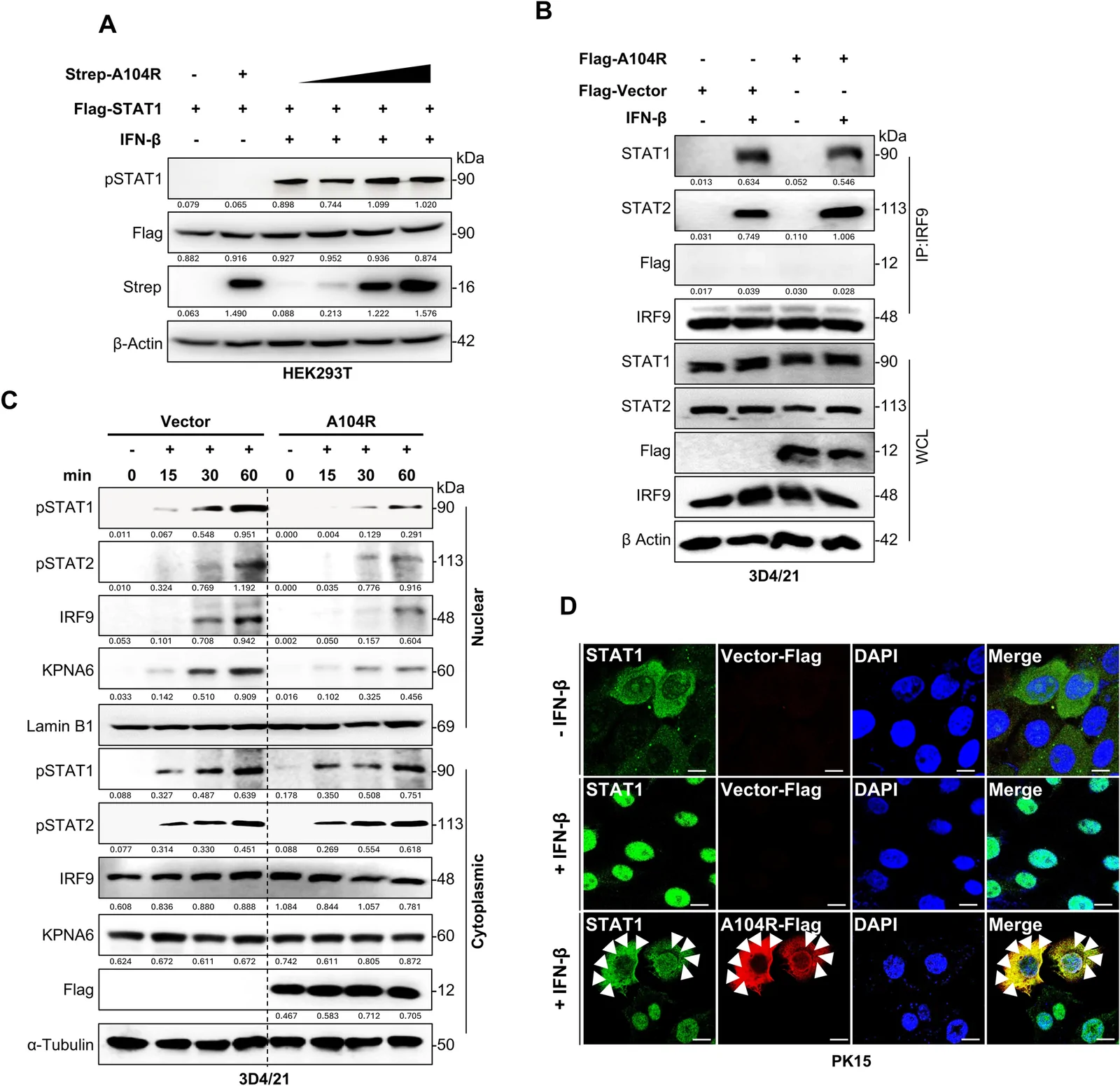
**A104R binding to KPNA6 does not alter STAT1 phosphorylation but reduces phosphorylated STAT1 nuclear transport.** **A** HEK293T cells were cotransfected with Flag-tagged STAT1 and increasing amounts of Strep-tagged A104R. At 36 hpt, cells were treated with IFN-β (1000 U/mL) for 1 h, and cell lysates were analyzed by immunoblotting. The numerical values below the panels represent the relative intensity normalized to the β-actin (internal control). **B** Cells were treated with IFN-β (1000 U/mL) for 2 h, lysed, and subjected to immunoprecipitation with IRF9 antibody to assess endogenous ISGF3 complex formation. The numerical values below the anti-STAT1, -STAT2, and -Flag panels in the IP: IRF9 represent the relative intensity of the STAT1, STAT2, and Flag-A104R signals, normalized to the IRF9 bait in the same IP: IRF9. **C** Stable 3D4/21 cells expressing Flag-A104R were treated with IFN-β at the indicated timepoints. Cytoplasmic and nuclear fractions were prepared using a Nuclear and Cytoplasmic Protein Extraction Kit and analyzed separately by immunoblotting. The numerical values below the panels in the nuclear and cytoplasmic fractions represent the relative intensity normalized to the internal controls of Lamin B1 and α-Tubulin, respectively. **D** Control- and Flag-A104R-expressing PK-15 cells were treated with IFN-β (1000 U/mL) at the indicated timepoints. Flag-A104R was detected using an anti-Flag antibody and a Cy3-conjugated secondary antibody. STAT1 and A104R are shown in green and red, respectively. Scale bar indicates 20 μm.

Type I and II IFN responses need STAT1 to be phosphorylated. To be more precise, type I IFNs promote the induction of ISRE genes, which results in an antiviral state and provides cell-intrinsic innate immunity. The inhibition of STAT1 phosphorylation was determined in the presence of A104R. We could not observe the specific inhibition of phosphorylation of STAT1 (Figure 7A). Nuclear translocation of STAT1 is critical for the signaling cascade induced by IFN-β and downstream gene expression [35]. After phosphorylation by JAK1 and tyrosine kinase 2 (Tyk2), the phosphorylated STAT1/STAT2 heterodimer interacts with IRF9 to form the ISGF3 complex [36, 37]. Since A104R does not directly bind to or inhibit the phosphorylation of STAT1, we next evaluated whether A104R affects ISGF3 formation. The 3D4/21 cells were transfected with Flag-A104R or a plasmid control and then mock-treated or treated with IFN-β. The cell lysates were co-immunoprecipitated using an antibody against IRF9, and the expression levels of p-STAT1, p-STAT2, and IRF9 were detected by immunoblotting. As shown in Figure 7B, overexpression of A104R did not affect the interactions between endogenous p-STAT1/p-STAT2 and IRF9. These results confirm that A104R overexpression does not influence ISGF3 formation. Once the ISGF3 complex is formed, it is translocated into the nucleus to initiate gene transcription of IFN stimulated genes (ISGs) by binding to ISRE [35, 38]. To investigate whether A104R affects the nuclear import of STAT1 induced by type I IFN. Here, 3D4/21 cells were transfected with A104R plasmids or an empty vector control for 24 h. After a 15, 30, and 60 min of treatment with IFN-β (1000 U/mL), nuclear and cytoplasmic fractionation assays were performed, followed by immunoblotting analysis with the relevant antibodies. The results showed that A104R significantly hindered the IFN-β-induced nuclear accumulation of STAT1 (Figure 7C). Next, PK-15 cells were transfected with the Flag-A104R expression plasmid, and the localization of STAT1 proteins in the presence of A104R was examined using a confocal microscope. In the absence of IFN-β treatment, STAT1 revealed that the protein was distributed in a diffuse pattern within cells. Following IFN-β treatment, there was a clear observation of STAT1 translocating from the cytoplasm to the nucleus, demonstrating that IFN-β induced nuclear accumulation of STAT1 in cells transfected with an empty vector. The expression of A104R failed to allow STAT1 to accumulate in the nucleus following IFN-β treatment (Figure 7D). Indicating that this translocation was inhibited by A104R, further confirming that A104R suppresses the nuclear translocation of STAT1.

### Inhibition of IFN-β-induced JAK/STAT signaling pathway by A104R

**Figure 8:**
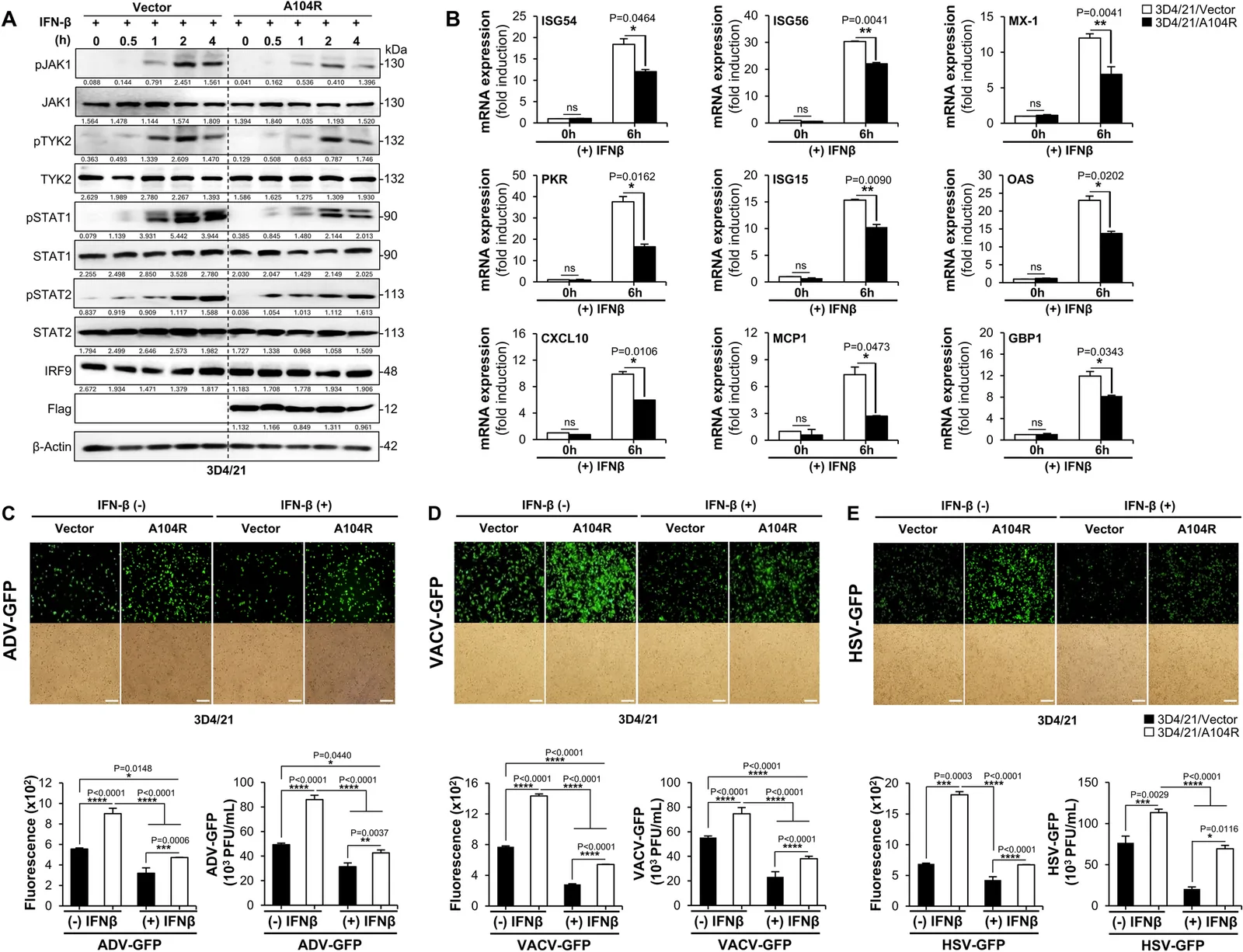
**A104R inhibits IFN-β-induced JAK/STAT signaling.** **A** Cells transfected with the vector control or A104R were treated with IFN-β for 0, 0.5, 1, 2, or 4 h. Phosphorylation of JAK1, TYK2, STAT1, and STAT2 was analyzed by immunoblotting. A104R expression was confirmed by detection of Flag-tagged A104R The numerical values below the panels represent the relative intensity normalized to the β-actin (internal control). **B** The 3D4/21 cells were transfected with Flag-tagged A104R or the control vector and treated with IFN-β for 2 h. At the indicated timepoints, total RNA was extracted and the mRNA levels of the indicated genes were measured by RT-qPCR. **C** The 3D4/21 cells expressing the control vector or A104R were infected with ADV-GFP in the absence or presence of IFN-β. GFP fluorescence indicates viral replication; fluorescence intensity and viral titers are shown below. **D** Cells transfected with the control vector or A104R were infected with VACV-GFP and treated with or without IFN-β. GFP fluorescence and quantitative analyses are shown. **E** Cells were transfected with the control vector or A104R and infected with HSV-GFP in the presence or absence of IFN-β. Fluorescence intensity and viral titers were determined. Data are representative of three independent experiments. Values represent the mean ± SD of three biological replicates from a representative experiment (**B**–**E**). Unpaired Student’s *t*-test was used for comparison between two independent groups (**B**). Significant group differences were analyzed one-way ANOVA followed by Tukey’s test (**C**–**E**). *P ≤ 0.05, **P ≤ 0.01, *** *P* ≤ 0.001, or **** *P* ≤ 0.0001. ns: not significant. Scale bar indicates 50 μm.

To further validate the results obtained, whether A104R, a viral protein, affects the antiviral effects of IFN-β by assessing the replication of various GFP-expressing viruses. The 3D4/21 cells stably expressing A104R or control vector were introduced with or without IFN-β (1000U/mL) and then infected with ADV-GFP (MOI = 1.0), VACV-GFP (MOI = 1.0), or HSV-GFP (MOI = 0.5). A104R expression significantly increases viral fluorescence, indicating enhanced replication compared with the vector control. This enhancement is particularly evident in the absence of IFN-β (left side of Figure 8C, D, E), where fluorescence levels are markedly higher in A104R-expressing cells. However, even in the presence of IFN-β (right side of Figure 8C, D, E), A104R continues to boost ADV-GFP replication, as shown by a significant increase in fluorescence. This data indicates that A104R can counteract the antiviral effects of IFN-β to some extent, allowing for greater virus replication. Quantification of viral titers further confirms that A104R expression significantly increases viral replication, particularly under conditions where IFN-β is present (bottom panel of Figure 8C, D, E), thereby demonstrating A104R’s role in modulating the antiviral response mediated by IFN-β. IFN-β induces a strong antiviral response by inducing the expression of ISGs, thus controlling viral infection [39]. To confirm the ability of pA104R to inhibit type I IFN signaling, the effect of pA104R on the induced transcription of endogenous ISGs was assessed. The 3D4/21 stably overexpressing A104R cells were treated with IFN-β, RT-qPCR using specific primers for the genes of interest (Table 2). The presence of pA104R significantly inhibited the induction of ISGs expression compared with that in the controls (Figure 8B). Subsequently, we examined the endogenous proteins and phosphorylation levels of these crucial molecules in the IFN-β-induced JAK/STAT signaling pathway. The 3D4/21 cells stably overexpressing A104R and control plasmids were treated with IFN-β for different lengths of time, and then the cell lysates were analyzed via immunoblotting. The phosphorylation levels of these proteins gradually decreased over time. In contrast, cells expressing A104R exhibited a marked reduction in the phosphorylation of JAK1, TYK2, STAT1, and STAT2 across all timepoints after IFN-β stimulation (Figure 8A). Taken together, these data suggest that A104R effectively inhibits the activation of the JAK/STAT pathway in response to IFN-β.

### Knockdown of pA104R reduces ASFV viral infection

**Figure 9:**
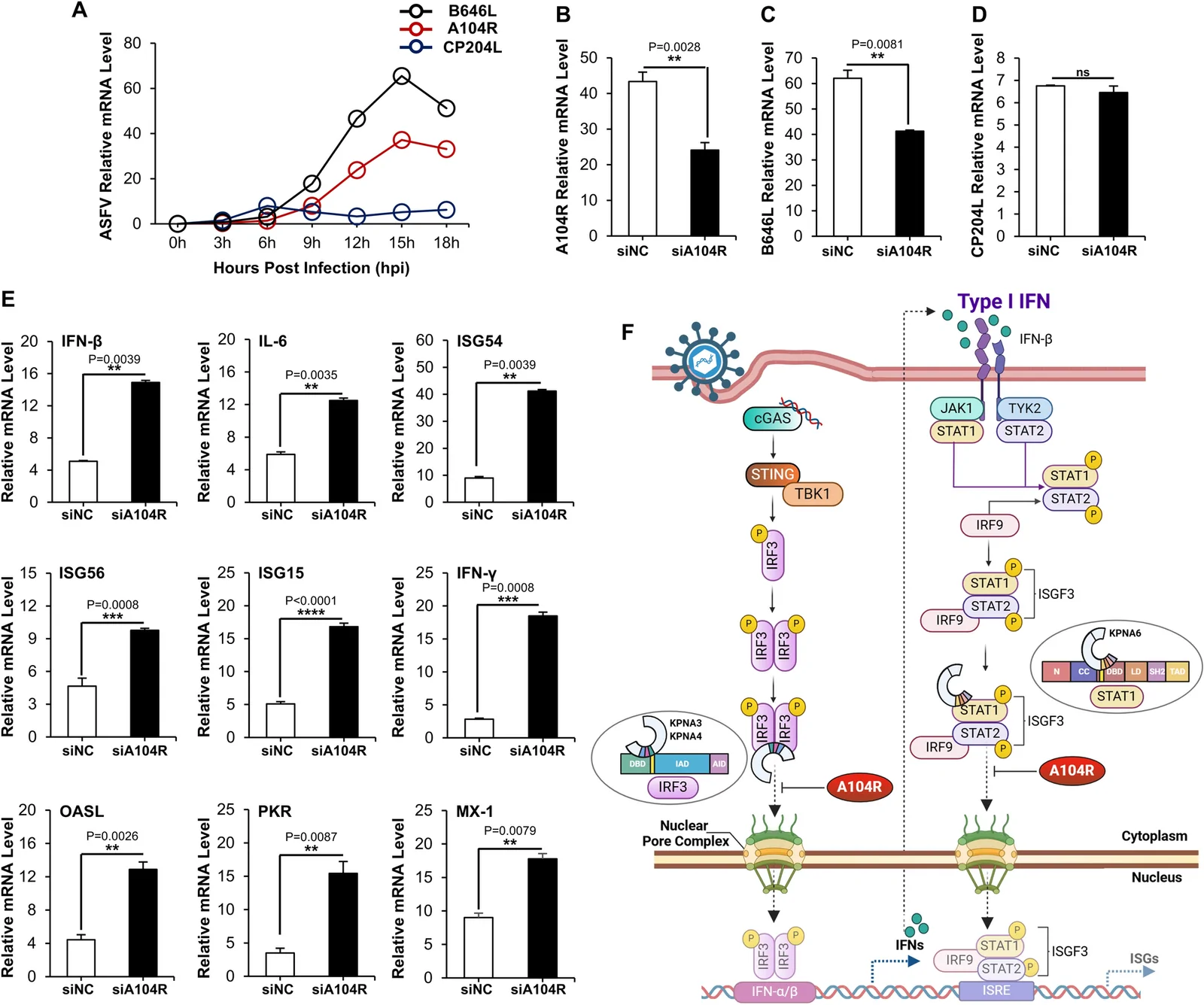
**A104R is a late-transcribed protein that promotes ASFV replication and antagonizes IFN and ISG transcription.** **A** Primary PAMs were infected with ASFV strain Korea/wild boar/Hwacheon/2020–2287 at an MOI of 0.5 and harvested at the indicated timepoints separately. Transcription of A104R, CP204L, and B646L was measured by RT-qPCR. p30 (CP204L) and p72 (B646L) served as markers of early and late viral gene expression, respectively. **B–E** Primary PAMs were transfected with either siRNAs targeting A104R or a control siRNA for 12 h and then infected with ASFV at an MOI of 0.5. Transcription of A104R (**B**), B646L (**C**), CP204L (**D**), and interferons (IFNs) and IFN stimulated genes (ISGs) (**E**) were detected at 16 hpi by RT-qPCR. **F** Graphical summary of the mechanism by which A104R suppresses the nuclear translocation of IRF3 and STAT1. Data are representative of three independent experiments. Values represent the mean ± SD of three biological replicates from a representative experiment (**B–E**). Unpaired Student’s *t*-test: *P ≤ 0.05, **P ≤ 0.01, *** *P* ≤ 0.001, or **** *P* ≤ 0.0001. ns: not significant.

To evaluate the transcriptional dynamics of A104R, RNA was extracted from primary PAMs that were infected with the ASFV strain Korea/wild boar/Hwacheon/2020–2287 at an MOI of 0.5. The mRNA levels of the viral genes were determined as previously described [40]. As illustrated in Figure 9A, the transcription pattern of A104R was very similar to that of B646L, a late-transcribed gene, but differed from early-transcribing CP204L gene. These findings support the conclusion that A104R is a late-transcribed gene of ASFV. To further explore the biological role of A104R, primary PAM cells were transfected with either siControl or siA104R and then infected with the ASFV strain Korea/wild boar/Hwacheon/2020–2287 at an MOI of 0.5. As expected, siA104R resulted in a reduced transcription of A104R of around 45% at 16 hpi (Figure 9B). Additional RT-qPCR analysis revealed lower transcription levels of ASFV B646L late gene in ASFV challenged siA104R cells (up to 33.5%) in comparison with siControl infected cells (Figure 9C). However, no significant difference in the transcriptional levels of CP204L early gene was observed (Figure 9D). Subsequently, upon A104R knockdown in ASFV-infected primary PAMs, the transcription levels of IFNs and ISGs were upregulated (Figure 9E). These findings revealed that A104R inhibits the transcription of IFN, leading to reduced ISGs and thus inducing viral replication. Taken together, the data demonstrate that A104R acts as a late-transcribed protein important in virus replication as well as involved in the suppression of IFNs and ISGs transcription.

## Discussion

ASFV employs a range of strategies to evade the host innate immune defenses, largely through complex interactions between viral proteins and host signaling pathways [41]. ASFV-encoded proteins suppress type I IFN responses and ISG expression, thereby promoting viral replication and persistence within the host [42].

For example, DP96R inhibits TBK1- and IKKβ-induced activation of the nuclear factor kappa light-chain-enhancer of activated B cells (NF-κB) promoter, reducing type I IFN production [43]. MGF505-3R promotes TBK1 degradation, thereby suppressing cGAS–STING-mediated IFN-β production [44], while MGF360-12L blocks IFN-β expression and ISRE activation by targeting cGAS–STING, TBK1, or IRF3-5D [45]. In addition, E120R disrupts IRF3 recruitment to TBK1, further inhibiting IRF3 activation and subsequent type I IFN expression [46]. MGF360-9L promotes the degradation of STAT1 and STAT2, effectively impairing IFN β signal transduction [47]. MGF505-7R interacts with STING to inhibit the cGAS–STING signaling pathway [48] and impair JAK1- and JAK2-mediated responses, thereby contributing to ASFV virulence and immune evasion [49].

In this study, we demonstrated a novel molecular mechanism underlying the inhibition of type I IFN signaling by A104R. First, GST pull-down coupled with mass spectrometry identified KPNA proteins as potential interacting partners of A104R and these interactions were further validated by IP and confocal microscopy. Second, domain mapping of KPNA4 revealed that A104R primarily interacts with its major NLS region, which overlaps with the IRF3-binding site. Competitive binding assays demonstrated that A104R interferes with the interaction between IRF3 and both KPNA3 and KPNA4. Third, we explored the functional consequences of A104R expression and found that A104R showed no effect on IRF3 phosphorylation or dimerization. However, cell fractionation and confocal microscopy confirmed that A104R effectively blocked the nuclear translocation of IRF3. Additional evidence supported this mechanism, showing that A104R expression increased viral replication and decreased IFN production, along with reduced IFN signaling and diminished expression of antiviral genes in various cell lines. Finally, we demonstrated that A104R also interacts with KPNA6, preventing the nuclear translocation of PY-STAT1 without affecting its upstream phosphorylation. Collectively, these results suggest that A104R employs a dual-targeted mechanism to impair both IRF3- and STAT1-mediated transcriptional responses, thereby suppressing type I IFN production and signaling.

Karyopherins, comprising KPNA and karyopherin-β (KPNB), are nuclear transport receptors that carry cargo proteins containing NLSs through nuclear pore complexes [50, 51]. Six human KPNA isoforms have been identified and classified into three distinct subfamilies based on cargo specificity and sequence similarity [52, 53]: the Rch1 subfamily (KPNA2), the Qip1 subfamily (KPNA3 and KPNA4), and the NPI-1 subfamily (KPNA1, KPNA5, and KPNA6) [53–59]. The three KPNA subfamilies share up to 50% sequence similarity. However, within the Qip1 subfamily, KPNA3 and KPNA4 share more than 85% sequence similarity, while the NPI-1 subfamily also shares more than 80% sequence similarity, reflecting a high degree of functional redundancy [34]. Structurally, KPNA proteins contain a central domain of ten tandem ARM repeat motifs organized into a superhelix, flanked by a small N-terminal importin β-binding domain and a C-terminal domain [52, 60–62]. ARM repeats 2–4, which form the major binding site and repeats 7–9, which constitute the minor binding site of KPNA bind cargo proteins containing classical NLSs such as IRF3 and STAT1. This interaction is essential for the nuclear translocation of both IRF3 and STAT1, which are central mediators of type I IFN responses [63–65]. Distinct ASFV proteins exploit several KPNA family proteins, suggesting a layered strategy to disrupt host nuclear transport. MGF360-12L targets KPNA2, KPNA3, and KPNA4, thereby disrupting IRF3 nuclear translocation and type I IFN induction [9]. In contrast, we demonstrated that A104R interacts with KPNA3, KPNA4, and KPNA6, thereby inhibiting the nuclear translocation of both IRF3 and STAT1.

Therefore, although both MGF360-12L and A104R target KPNA3 and KPNA4, which are closely related Qip1 subfamily members, they can inhibit innate immunity complementarily due to their different KPNA-binding profiles.

MGF360-12L may restrict early IRF3-dependent IFN production, whereas A104R may extend this inhibition by blocking IRF3 and STAT1-dependent IFN production. Targeting of KPNA6 by A104R further suggests that ASFV expands its inhibition to multiple KPNA subfamilies to overcome the cargo-transport functions among KPNA proteins. Overlapping activities targeting KPNA can enhance pathogenicity by inhibiting antiviral signaling in different cellular environments and stages.

Recently, specific viral proteins that target these nuclear transport receptors to evade innate immune activation have been identified. For instance, Japanese encephalitis virus NS5 targets KPNA3 and KPNA4 to prevent the nuclear translocation of IRF3 and NF-κB [23]. Hepatitis C virus NS3/4A protein restricts IRF3 and NF-κB translocation by cleaving KPNB1 [66]. Vaccinia virus protein A55 interacts with KPNA2, disrupting its interaction with NF-κB [67]. ASFV MGF360-12L targets KPNA2–4 to prevent IRF3 and NF-κB nuclear translocation [9]. In addition to IRF3 and NF-κB, several viruses interfere with the IFN signaling pathway by blocking various aspects of STAT1 activation and nuclear translocation [68]. Ebola virus VP24 binds to KPNA1 and blocks STAT1 nuclear accumulation [69]. Porcine reproductive and respiratory syndrome virus (PRRSV) NSP1β inhibits interferon-activated JAK/STAT signal transduction by inducing KPNA1 degradation [70]. Porcine epidemic diarrhea (PED) virus nsp7 hinders the interferon-triggered JAK/STAT signaling pathway by blocking the interaction between KPNA1 and STAT1 [71]. Ebola virus VP24 also targets a KPNA5 NLS-binding site to specifically inhibit the nuclear import of PY-STAT1 [33].

In this study, we demonstrated that KPNA3, KPNA4, and KPNA6 are potential binding partners of A104R through GST pull-down experiments and mass spectrometry. Domain mapping results using truncated forms of KPNA4 revealed that both A104R and IRF3 specifically interact with the major NLS-binding site within the KPNA4 ARM repeat motif (repeats 2–4) and that A104R inhibits IRF3–KPNA interaction in a dose-dependent manner. This suggests that A104R competitively binds to KPNA3 and KPNA4, thereby preventing IRF3 association. Importantly, A104R did not affect IRF3 phosphorylation or dimerization. Instead, cell fractionation and confocal microscopy revealed that A104R specifically blocks the nuclear translocation of IRF3, thereby preventing its transcriptional activation of the IFN-β promoter and subsequent antiviral gene expression. The results suggest that A104R selectively hijacks KPNA3, KPNA4, and KPNA6 to impair the nuclear import of IRF3 and STAT1.

In addition, an essential step within the JAK/STAT signaling pathway involves the phosphorylation and nuclear translocation of PY-STAT1, a process mediated by members of the NPI-1 subfamily of KPNA (KPNA1, KPNA5, and KPNA6) [31, 32]. We also found the interaction between A104R and KPNA6 (Figures 3C and 6A). In previous studies, PY-STAT1 is known to interact with the C-terminal ARM repeats of KPNA1 and KPNA5 during nuclear import [33, 72]. Therefore, we hypothesized that A104R could similarly target the C-terminal region of KPNA6. Consistent with this hypothesis, our domain mapping study revealed that A104R binds to ARM repeats 7–10 of KPNA6 and that A104R inhibits PY-STAT1–KPNA interaction in a dose-dependent manner.

The temporal expression pattern of A104R indicates the functional advantage during ASFV infection. Predominantly, A104R is expressed during the late phase of ASFV infectious cycle, coinciding with the viral genome replication, late-gene transcription, and progeny virion assembly [21, 72].

Infected cells and neighboring bystander cells are exposed to increasing levels of type I IFNs and other inflammatory mediators [73]. Although early expressing ASFV proteins can suppress pathogen-sensing and antiviral pathways, such as cGAS-STING signaling. A104R could simultaneously impair continuous IFN transcription and cellular response to IFNs in ASFV-infected cells by competitively preventing IRF3 import by KPNA3/KPNA4 and phosphorylated STAT1 import by KPNA6. This dual mechanism limits both autocrine and paracrine amplification of the antiviral state, thereby ensuring conditions favorable for late viral replication. Taken together, A104R may function as an immune safeguard at the late phase of ASFV infection that complements ASFV proteins acting at the early phase. Nuclear import mediated by KPNAs is a conserved and key host process used by antiviral transcription factors [74, 75]. ASFV appears to exploit conserved KPNA family proteins to inhibit nuclear accumulation of IRF3 and STAT1. Such a strategy provides robustness against functional inhibition of related KPNA import receptors and thereby impaired IFN production. Binding profiles of evolutionary conserved KPNAs with A104R suggest an evolutionary strategy in ASFV to keep suppressed antiviral transcription during viral infection.

Chen et al. reported that A104R inhibits IFN-α-induced STAT1 phosphorylation in HEK293T cells at late timepoints [76]. In contrast, we could not observe the inhibition in STAT1 phosphorylation following IFN-β stimulation in HEK293T cells and, porcine 3D4/21 and PK-15 cells. Instead, our findings support a mechanism in which A104R preferentially and competitively binds to components of the PY-STAT1 nuclear import machinery without affecting the formation of ISG factor 3 complexes (Figure 7B). Despite these differences, both studies converge on the important point that A104R does not impair ISGF3 complex assembly. Our KPNA6-competition model offers a mechanistically defined explanation that complements the phenotypic observation of impaired IFN signaling reported previously, extending the current understanding of how A104R antagonizes antiviral defense in the host.

Deletion of A104R from the highly virulent Georgia isolate markedly reduced virulence in domestic pigs, with remaining animals clinically healthy and developing robust antibody responses [22]. These findings show that A104R is an important virulence determinant. Mechanistically, loss of A104R could impose a multifaceted attenuation of ASFV by impaired viral histone-like protein function, as well as restoring host IRF3 and STAT1 antiviral responses. Further, attenuation in the A104R-deficient SY18 isolate was limited, highlighting that the phenotypic cost of A104R deletion may vary depending on the virus strain or isolate [77]. Therefore, deletion of A104R is an effective candidate for multigene-deletion vaccine platforms compared with a single-gene deletion vaccine. A104R deletion could be combined with the deletion of other nonessential virulent genes to enhance vaccine safety and efficacy while preserving enough viral replication to induce protective humoral and cellular immune responses.

Our study proposes a model in which the A104R antagonizes host innate immune signaling by disrupting the nuclear import of key transcription factors. Specifically, A104R impedes IRF3 nuclear import by competitively engaging KPNA3 and KPNA4 and blocks STAT1 nuclear import by recruiting KPNA6 (Figure 9F). This dual inhibition effectively blocks the host’s antiviral response by inhibiting the type I IFN signaling pathway. A104R is essential for ASFV replication [21] and deletion of B125R from the ASFV-SY18 strain caused a frameshift mutation in A104R, resulting in a recombinant virus exhibiting slightly attenuated virulence in domestic pigs [77]. Interestingly, a recombinant virus with the A104R gene removed from the Georgia isolate significantly reduced pathogenicity in domestic pigs [22]. This attenuation is very likely due to the loss of A104R-mediated antiviral response suppression.

## Supplementary Information

**Additional file 1. Screening of the ASFV gene library based on ADV-GFP replication. **PK-15 cells were transfected with the control vector or HA-tagged plasmids containing 158 ASFV genes cloned into pIRES-HA (GenBank accession no. FR682468.1). At 24 hpt, cells were infected with ADV-GFP at an MOI of 1. At 24 hpi, viral replication was examined by fluorescence microscopy and quantified using a fluorescence modulator.

**Additional file 2.** **Overexpression and structural modeling confirm the interaction between A104R and KPNA3/KPNA4. A** Stable 3D4/21 cells expressing Flag-A104R were harvested and subjected to anti-Flag immunoprecipitation and immunoblotting with anti-IRF3 and anti-Flag antibodies. The numerical values below the anti-Flag panel in the IP: Flag represent the relative intensity of the IRF3 signal, normalized to the corresponding Flag bait in the same IP: Flag. **B** HEK293T cells were transfected with Flag-tagged A104R for 36 h, followed by anti-Flag immunoprecipitation and immunoblotting with anti-KPNA3 and anti-Flag antibodies. The numerical values below the anti-KPNA3 panel in the IP: Flag represent the relative intensity of the KPNA3 signal, normalized to the corresponding Flag bait in the same IP: Flag. **C** HEK293T cells were transfected with Strep-tagged A104R for 36 h, followed by Strep pull-down and immunoblotting with the indicated antibodies. The numerical values below the anti-KPNA4 panel in the Strep PD represent the relative intensity of the KPNA4 signal, normalized to the corresponding Strep bait in the same Strep PD. **D** AlphaFold3 structural modeling was used to predict the interaction interfaces between A104R (Gene Bank: FR682468.1) and KPNA3 (Uniprot ID: A0A5G2R9E6_PIG). The 3D surface model shows strong interaction between A104R and KPNA3. **E** The ribbon diagram shows the interacting residues between A104R and KPNA3 with respective distances.

**Additional file 3. Overexpression rescue assay confirms recovery of IRF3-KPNA3 and -KPNA4 interaction. A**, **B** 3D4/21 cells were co-transfected with Strep-A104R with increasing amounts of Flag-KPNA3 or GST-KPNA4 plasmids before ADV-GFP infection. Cell lysates were subjected to Flag immunoprecipitation or GST pulldown. The recovery of IRF3-KPNA3 interaction was revealed by immunoblotting with anti-IRF3 and anti-Flag antibodies. The numerical values below the anti-IRF3 and -Strep panels in the IP: Flag represent the relative intensity of the IRF3 and Strep-A104R signals, normalized to the corresponding Flag-KPNA3 bait in the same IP: Flag (**A**). The recovery of IRF3-KPNA4 interaction was revealed by immunoblotting with anti-IRF3 and anti-GST antibodies. The numerical values below the anti-IRF3 and -Strep panels in the GST PD represent the relative intensity of the IRF3 and Strep-A104R signals, normalized to the corresponding GST-KPNA4 bait in the same GST PD (**B**).

**Additional file 4. A104R-mediated inhibition of antiviral gene transcription. A, B** 3D4/21 (**A**) and PK-15 (**B**) cells were transfected with Flag-tagged A104R or the control vector and infected with ADV-GFP at an MOI of 1 at 24 hpt. At the indicated time points, total RNA was extracted and the mRNA levels of the indicated genes were measured by RT-qPCR. Data are representative of three independent experiments. Values represent the mean ± SD of three biological replicates from a representative experiment. Unpaired Student’s *t*-test: *P ≤ 0.05, **P ≤ 0.01, *** *P* ≤ 0.001 or **** *P* ≤ 0.0001.

**Additional file 5.** **Protein expression of overexpressed A104R. **A104R protein expression in 3D4/21 (**A**), PK-15 (**C**), and MA-104 (**E**) cells and A104R transcription in 3D4/21 (**B**), PK-15 (**D**), and MA-104 (**F**) cells were examined. Cells were transfected with the control vector or Flag-tagged A104R. Protein expression was analyzed by immunoblotting. The numerical values below the panels represent the relative intensity normalized to the β-actin (internal control). Total RNA was extracted for cDNA synthesis followed by RT-qPCR.

**Additional file 6. A104R negatively regulates antiviral immune responses in PK-15 and MA-104 cells. **PK-15 cells were transfected with Flag-tagged A104R or the control vector and infected with ADV-GFP (**A**) or VACV-GFP (**B**) at an MOI of 1 at 24 hpt. Viral replication was visualized by GFP fluorescence microscopy at 24 hpi and quantified at 12 and 24 hpi. Viral titers were determined by plaque assay using A549 cells. Porcine IFN-β and IL-6 concentrations in culture supernatants were measured by ELISA (**E, F**). MA-104 cells stably expressing Flag-tagged A104R or the control vector were infected with ADV-GFP (**C**) or VACV-GFP (**D**) at an MOI of 1 at 24 hpt. Viral replication was assessed and quantified as described above, and viral titers were determined by plaque assay using A549 cells. Human IFN-β and IL-6 concentrations in culture supernatants were measured by ELISA (**G**, **H**). Data are representative of three independent experiments. Values represent the mean ± SD of three biological replicates from a representative experiment. Unpaired Student’s *t*-test: *P ≤ 0.05, **P ≤ 0.01, *** *P* ≤ 0.001 or **** *P* ≤ 0.0001. Scale bar indicates 50 μm.

**Additional file 7.** **Structural modeling confirms the interaction between A104R and KPNA6. A** AlphaFold3 structural modeling was used to predict the interaction interfaces between A104R (Gene Bank: FR682468.1) and KPNA6 (Uniprot ID: A0A4X1VYA1_PIG). The 3D surface model shows strong interaction between A104R and KPNA6. **B** The ribbon diagram shows the interacting residues between A104R and KPNA6 with respective distances.

## Data Availability

No datasets were generated or analyzed during the current study.

## Abbreviations

A104R/pA104R: ASFV putative histone-like protein A104R
ADV-GFP: Green fluorescent protein-expressing adenovirus
ANOVA: Analysis of variance
ARM repeats: Armadillo repeats
ASF: African swine fever
ASFV: African swine fever virus
BSA: Bovine serum albumin
BSL-3: Biosafety level 3
cDNA: Complementary DNA
cGAMP: Cyclic GMP–AMP (dinucleotide)
cGAS: Cyclic GMP–AMP synthase
DAPI: 4′,6-Diamidino-2-phenylindole
ECL: Enhanced chemiluminescence
ELISA: Enzyme-linked immunosorbent assay
FBS: Fetal bovine serum
GAS: IFN-gamma-activated site
GST: Glutathione S-transferase
HEK293T: Human embryonic kidney 293 T cell line
hpi: Hours post-infection
hpt: Hours post-transfection
HRP: Horseradish peroxidase
HSV-GFP: GFP-expressing herpes simplex virus
IFIT1: Interferon-induced protein with tetratricopeptide repeats 1
IFITM3: Interferon-induced transmembrane protein 3
IFN: Interferon
IFN-β: Interferon-beta
IFN-γ: Interferon-gamma
IKKε: Inhibitor of nuclear factor kappa B kinase subunit epsilon
IL-1β: Interleukin 1 beta
IL-6: Interleukin 6
IP: Immunoprecipitation
IRF3: Interferon regulatory factor 3
IRF9: Interferon regulatory factor 9
ISG: Interferon-stimulated gene
ISG-15: Interferon-stimulated gene 15
ISGF3: Interferon-stimulated gene factor 3
ISRE: Interferon-stimulated response element
IκBα: Inhibitor of nuclear factor kappa B, alpha
JAK: Janus kinase
JAK1: Janus kinase 1
KPNA: Karyopherin-alpha
KPNA1–KPNA6: Karyopherin-alpha isoforms 1 to 6
KPNB: Karyopherin-beta
MA-104: Monkey kidney epithelial cell line
MCP-1: Monocyte chemoattractant protein 1
MOI: Multiplicity of infection
mRNA: Messenger RNA
MX-1: MX dynamin-like GTPase 1
nd: Not detected
ns: Not significant
NF-κB: Nuclear factor kappa-light-chain-enhancer of activated B cells
NIWDC: National Institute of Wildlife Disease Control and Prevention
NLS: Nuclear localization signal
NPC: Nuclear pore complex
OASL: 2'-5'-Oligoadenylate synthetase-like protein
p65: RELA proto-oncogene, NF-κB subunit p65 (RELA)
PAM: Primary porcine alveolar macrophage
PAMP: Pathogen-associated molecular pattern
PBS: Phosphate-buffered saline
PBST: Phosphate-buffered saline with Tween-20
PED virus: Porcine epidemic diarrhea virus
PEI: Polyethyleneimine
PFU: Plaque-forming unit(s)
PK-15: Porcine kidney-15 cell line
PKR: Interferon-induced double-stranded RNA-activated protein kinase
PRR: Pattern recognition receptor
PRRSV: Porcine reproductive and respiratory syndrome virus
PVDF: Polyvinylidene difluoride (membrane)
PY-STAT1: Phosphorylated STAT1
RT-qPCR: Quantitative real-time polymerase chain reaction
RIPA: Radio-immunoprecipitation assay (lysis buffer)
SD: Standard deviation
SDS-PAGE: Sodium dodecyl sulfate-polyacrylamide gel electrophoresis
SeV: Sendai virus
siRNA: Small interfering RNA
SOP: Standard operating procedure
STAT1: Signal transducer and activator of transcription 1
STAT2: Signal transducer and activator of transcription 2
STING: Stimulator of interferon genes
TBK1: TANK-binding kinase 1
TBST: Tris-buffered saline with Tween-20
TNF-α: Tumor necrosis factor alpha
TYK2: Tyrosine kinase 2
Type I IFN/IFN-I: Type I interferon
VACV-GFP: GFP-expressing vaccinia virus
WCL: Whole-cell lysate
